# An intronic LINE-1 regulates IFNAR1 expression in human immune cells

**DOI:** 10.1186/s13100-023-00308-3

**Published:** 2023-11-30

**Authors:** Carmen A. Buttler, Daniel Ramirez, Robin D. Dowell, Edward B. Chuong

**Affiliations:** Department of Molecular, Cellular, and Developmental Biology and BioFrontiers Institute, University of Colorado Boulder, Boulder, CO 80309 USA

**Keywords:** LINE-1, Transposons, Enhancer, Interferon

## Abstract

**Background:**

Despite their origins as selfish parasitic sequences, some transposons in the human genome have been co-opted to serve as regulatory elements, contributing to the evolution of transcriptional networks. Most well-characterized examples of transposon-derived regulatory elements derive from endogenous retroviruses (ERVs), due to the intrinsic regulatory activity of proviral long terminal repeat regions. However, one subclass of transposable elements, the Long Interspersed Nuclear Elements (LINEs), have been largely overlooked in the search for functional regulatory transposons, and considered to be broadly epigenetically repressed.

**Results:**

We examined the chromatin state of LINEs by analyzing epigenomic data from human immune cells. Many LINEs are marked by the repressive H3K9me3 modification, but a subset exhibits evidence of enhancer activity in human immune cells despite also showing evidence of epigenetic repression. We hypothesized that these competing forces of repressive and activating epigenetic marks might lead to inducible enhancer activity. We investigated a specific L1M2a element located within the first intron of Interferon Alpha/Beta Receptor 1 (IFNAR1). This element shows epigenetic signatures of B cell-specific enhancer activity, despite being repressed by the Human Silencing Hub (HUSH) complex. CRISPR deletion of the element in B lymphoblastoid cells revealed that the element acts as an enhancer that regulates both steady state and interferon-inducible expression of IFNAR1.

**Conclusions:**

Our study experimentally demonstrates that an L1M2a element was co-opted to function as an interferon-inducible enhancer of IFNAR1, creating a feedback loop wherein IFNAR1 is transcriptionally upregulated by interferon signaling. This finding suggests that other LINEs may exhibit cryptic cell type-specific or context-dependent enhancer activity. LINEs have received less attention than ERVs in the effort to understand the contribution of transposons to the regulatory landscape of cellular genomes, but these are likely important, lineage-specific players in the rapid evolution of immune system regulatory networks and deserve further study.

**Supplementary Information:**

The online version contains supplementary material available at 10.1186/s13100-023-00308-3.

## Background

Transposable elements litter the genomes of eukaryotes, constituting over 50% of the human genome [1]. These transposable elements were historically considered to be strictly parasitic in nature, transcribing and re-integrating many copies of themselves throughout their host genome, and sometimes harming the host when their integration damages coding genes or interrupts regulatory networks [2, 3]. More recently, it has become increasingly appreciated that many transposable elements have acquired functions beneficial to their host during the course of their co-evolution, notably including functions as enhancers and other cis-regulatory elements [4–9].

In mammals, nearly all characterized examples of transposon-derived regulatory elements are derived from endogenous retroviruses (ERVs). Over time, most ERVs in the human genome have lost their protein coding regions through truncation and recombination, leaving behind the Long Terminal Repeat (LTR) elements. Retroviral LTRs are often rich with transcription factor binding sequences that originally evolved to hijack the host transcriptional machinery to promote proviral transcription [8, 10, 11]. As ERVs replicate throughout host genomes, they disperse a potential source of “ready-made” regulatory elements that can be co-opted to form cellular regulatory networks [6].

Long Interspersed Line Elements (LINEs) are another subclass of transposable elements, and are the only class capable of autonomous transposition in the human genome [12]. Given the potential threats posed by active transposition, it is unsurprising that they are specifically targeted by their host cells for transcriptional repression. The Human Silencing Hub (HUSH) complex has been observed to target transcribed transposons, prominently including LINEs, for epigenetic repression by driving the deposition of repressive trimethylation marks at the lysine 9 residue of histone 3 (H3K9me3) [13–15]. Unlike ERVs, LINEs do not contain LTRs, but they do contain 5’ untranslated regions that function as both sense and antisense promoters, as well as binding motifs for regulatory transcription factors, such as YY1 [16]. However, LINE retrotransposition is often truncated at the 5’ end [17]. This tendency to truncate the regulatory elements, along with the targeted epigenetic repression of LINEs makes them a less obvious source of cis-regulatory enhancer activity, but studies have explored other mechanisms by which LINEs have been shown to regulate expression of host genes [16]. For instance, LINEs have been observed to regulate their host genes through transcriptional interference [14, 18], as non-coding RNAs [19], and as a source of immunostimulatory RNA [20–22]. Multiple studies have predicted LINE enhancer activity based on epigenomic data [23–27], but their biological significance remains uncharacterized.

In this study, we investigated the epigenetic status of LINEs in the context of immune gene regulation by analyzing publicly available chromatin profiling data from human primary blood cells [28] and a lymphoblastoid cell line [29]. We found that while most LINEs exhibit repressive H3K9me3 marks, a significant subset also show active marks such as the acetylation of the lysine 27 residue of histone 3 (H3K27ac) and the monomethylation of the lysine 4 residue (H3K4me1) [30, 31]. Interestingly, in some cases, these activating, enhancer-associated modifications overlap with repressive H3K9me3 at the same LINEs. Other similar patterns of bivalent active and repressive epigenetic marks have been noted in undifferentiated stem cells and are proposed to act as primed enhancers, ready to be rapidly activated upon signaling [32, 33]. Rapid inducible transcriptional responses are also important in immune signaling, and we hypothesized that these bivalent LINEs may contribute similarly inducible enhancer elements.

We chose one such instance to study more closely: a LINE insertion of the L1M2a family within the first intron of the Interferon Alpha/Beta Receptor 1 (IFNAR1) gene. The 5’ end of this element appears to be intact, but the 3’ end is truncated, rendering it transpositionally inactive. It is nevertheless silenced by the HUSH complex [14], and yet exhibits both epigenetic and nascent transcriptional markers of potential enhancer activity. IFNAR1, the most likely target gene of any regulatory activity the intronic LINE possesses, is a key component of the type I interferon (IFN) receptor complex. Signaling through this complex drives a massive program of transcriptional changes needed to prepare cells to mount an immune response and defend against pathogens and other threats, so the proper regulation of the receptor components is pivotal to the proper function of the innate immune response [34]. We judged the intronic LINE, which we dubbed “IFNAR1.L1M2a,” to be a good candidate for experimental clarification of the possible roles of LINE-derived regulatory elements in the human immune system. Using CRISPR knockouts, we found that the IFNAR1.L1M2a element acts as an enhancer of IFNAR1 expression, modulating both steady state IFNAR1 transcription as well as the upregulation of the gene during IFN signaling.

## Results

### A subset of LINE insertions exhibit both repressive and active epigenetic marks

**Fig. 1:**
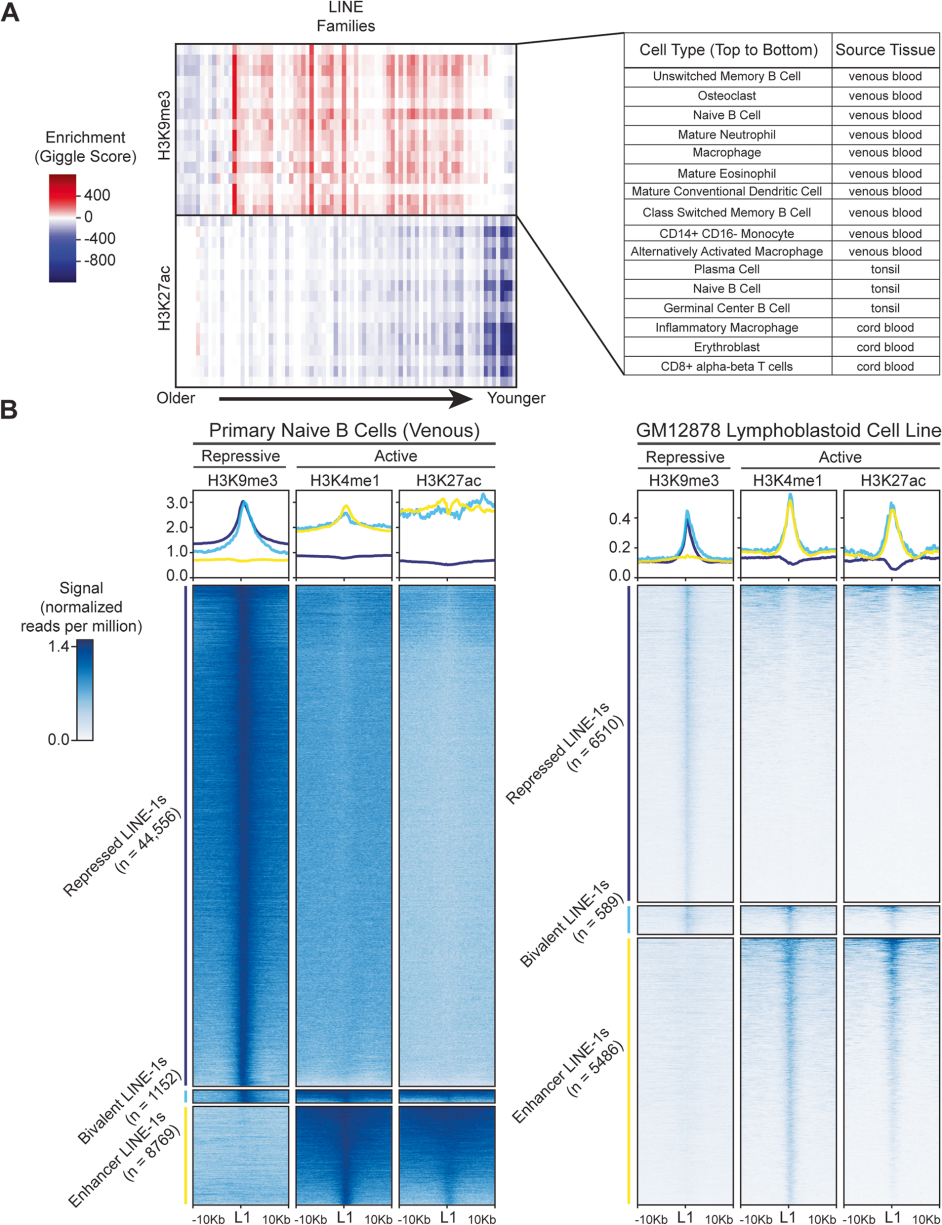
LINEs are predominantly epigenetically repressed, but a subset also exhibit enhancer-associated epigenetics. **A** A heatmap shows enrichment (red) and depletion (blue) of LINE families for the histone modifications H3K9me3 (top) and H3K27ac (bottom) in 16 publicly available human primary blood cell types obtained through the Blueprint Database [28]. Enrichment of ChIP signal at LINEs was quantified using Giggle [38]. Columns represent annotated LINE families with enrichment scores of more than 100 or depletion scores of less than -100 for either histone mark in any of the datasets shown. Unfiltered heatmaps of all LINE families, as well as SINE, ERV and DNA transposon families are available in (Additional file 1: Fig. S1). Most LINE families, especially younger L1M and L1P families are strongly enriched for repressive H3K9me3 and strongly depleted for activating H3K27ac, as expected. **B** Heatmaps and metaplots show ChIP signal of repressive H3K9me3 and enhancer associated H3K4me1 and H3K27ac at individual LINE-1s of lengths > 500 bp, in both primary naive B cells [28], and in the lymphoblastoid cell line GM12878 [29]. 10 kb windows were set around the 5’ end of the LINE-1 to cover the entire length of LINEs. Many LINE-1s are silenced by H3K9me3 as expected (navy), and some have enhancer associated epigenetic modifications (yellow). A subset, labeled as “bivalent” exhibit both repressive and enhancer associated signals (cyan)

To investigate the epigenomic features of LINE elements, we examined publicly available chromatin immunoprecipitation (ChIP) sequencing datasets from primary human immune cells [28]. We confirmed that LINE families are globally enriched within regions marked by the repressive H3K9me3 modification, and depleted within regions marked by the enhancer-associated H3K27ac modification, particularly younger LINE families (Fig. 1A). These results are in agreement with previous studies that showed specific H3K9me3-mediated silencing of LINEs in differentiated cells [13–15, 35]. A similar pattern of enrichment for H3K9me3 and depletion of H3K27ac was observed among ERV families (Additional file 1: Fig. S1), which are also subject to H3K9me3-mediated silencing [13, 36]. In previous studies, candidate ERV insertions that might possess enhancer activity have been identified by looking for overlap of ERV sequences with enhancer associated markers, including H3K27ac as well as H3K4me1 [23, 37]. In order to identify LINE insertions that potentially exhibit enhancer activity, we looked for individual LINEs that overlap H3K27ac and/or H3K4me1 peaks, both in primary naive B cells [28] and in the human lymphoblastoid cell line GM12878 [29] (Fig. 1B, Additional file 2: Table S1, Additional file 3: Table S2). In primary cells, we found a fraction (~ 13.9%, 44,556) of LINE-1 insertions to be repressed by H3K9me3 and a smaller fraction (~ 3.1%, 8769) with markers of potential enhancer activity (Fig. 1B, Additional file 1: Fig. S2A). Fewer LINEs showed epigenetic modifications in ChIP-Seq data from the lymphoblastoid cell line than in the primary cells, but the repressive H3K9me3 was still most abundant (~ 2.2%, 6510), with a smaller number of enhancer-like elements (~ 1.9%, 5486) (Fig. 1B, Additional file 1: Fig. S2A).

Interestingly, we identified a subset of LINE insertions that showed both repressive and activating marks. A fraction (in primary cells ~ 0.36%, 1152; in lymphoblastoid cell line ~ 0.18%, 589) of LINE-1s overlapped both repressive H3K9me3 peaks and enhancer associated peaks (Fig. 1B, Additional file 1: Fig. S2A), reminiscent of bivalent H3K27me3/H3K4me3 domains documented previously in embryonic stem cells [33, 39]. Both H3K9me3 silencing and these overlapping signals were considerably less abundant among the older LINE-2 elements (Additional file 1: Fig. S2B), consistent with the idea that epigenetic repression is primarily targeted against young transposons [40].

LINE-1s marked by enhancer associated markers, or by both H3K9me3 and enhancer associated markers (here called “bivalent”), but not those marked by H3K9me3 only, were enriched near immune genes in immune cells, suggesting a possible regulatory role (Additional file 1: Fig. S3). Genes proximal to the bivalently marked LINE-1s in naive B cells include multiple IFNs, interleukins, cytokine receptors and immune-related transcription factors (Additional file 4: Table S3).

The pattern of bivalent active and repressive marks seen in bulk datasets could be indicative of primed inducible activity [33]. We sought to investigate this possible novel function of LINEs by characterizing an example locus.

### IFNAR1.L1M2a is an example of a bivalently-marked LINE

**Fig. 2:**
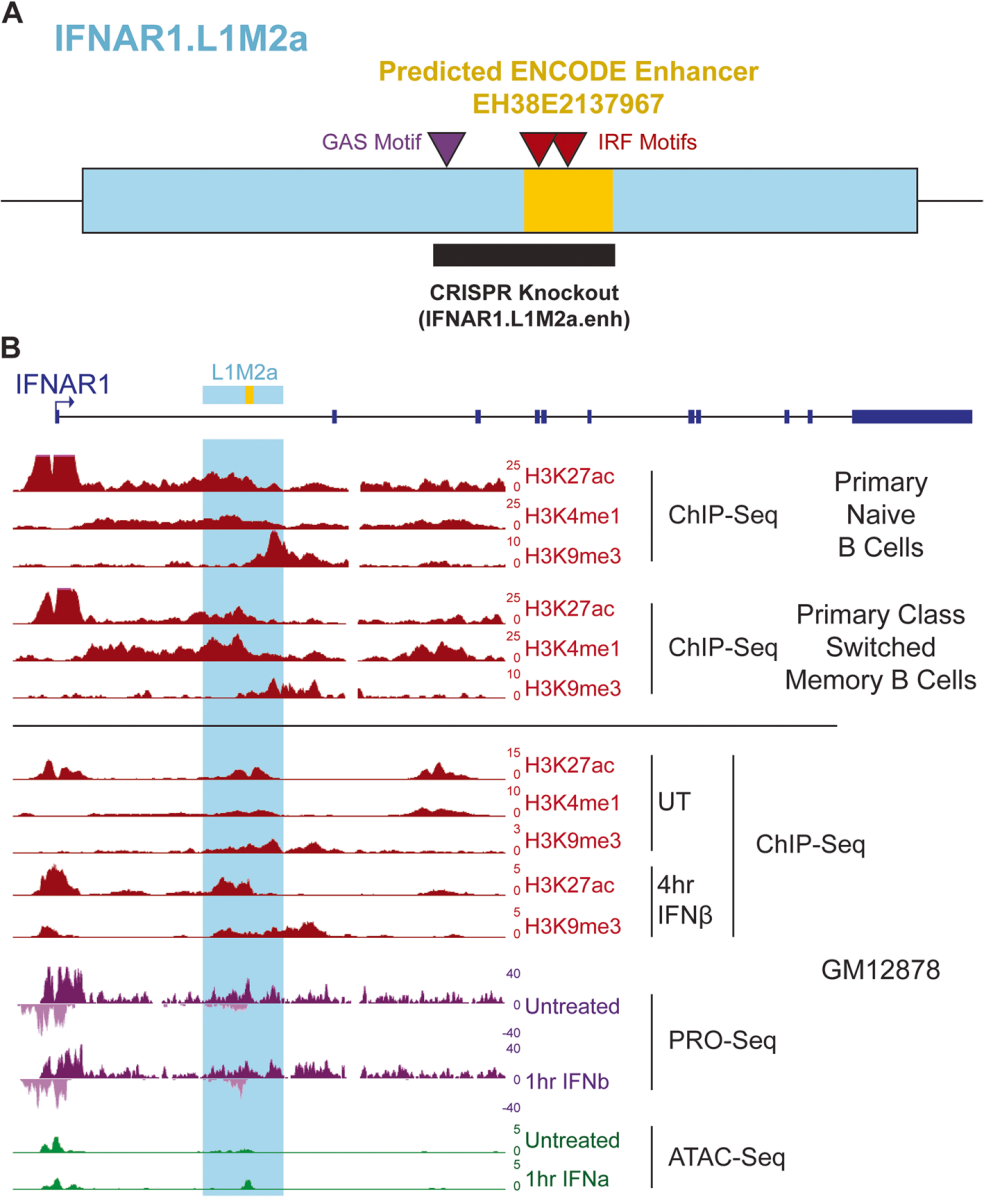
IFNAR1.L1M2a was chosen as an example locus for experimental exploration. **A** IFNAR1.L1M2a (blue) contains a predicted distal enhancer element identified by the ENCODE consortium (orange) [43]. Within or nearby this predicted enhancer region are several immune-related transcription factor binding motifs (red, purple). We sought to knock out this potential enhancer region, dubbed IFNAR1.L1M2a.enh (black). **B** The IFNAR1 gene (navy) locus. Publicly available ChIP-seq data (red), obtained from the Blueprint Database [28] and ENCODE consortium [29], along with our CUT&Tag data, describing the epigenetic landscape at the IFNAR1.L1M2a locus. Naive B cells, class switched memory B cells and the B cell-like GM12878 cell line exhibit bivalent repressive and enhancer associated histone modifications across the L1M2a insertion (highlighted blue). PRO-seq (purple) and ATAC-seq (green) both support the presence of an enhancer at the predicted site in IFNAR1.L1M2a, potentially induced by type I IFN treatment

We identified a L1M2a element within the first intron of the Interferon Alpha/Beta Receptor 1 (IFNAR1) gene that is marked by HUSH-dependent H3K9me3 [14], but exhibits bivalent epigenetic repressive and active markers in a subset of immune cell types including B cells (Fig. 2, Additional file 1: Fig. S4). The IFNAR1 gene encodes one of the two subunits of the type I IFN receptor complex which transduces extracellular IFN signaling into an intracellular transcriptional response, including the induction of many Interferon Stimulated Genes (ISGs) necessary for mounting a cellular immune response [41]. This LINE, which we refer to as IFNAR1.L1M2a, is conserved throughout Catarrhini [42] (Additional file 1: Fig. S4). We also observed ATAC-seq and PRO-Seq signals at the IFNAR1.L1M2a locus (GSE217294) (Fig. 2), consistent with enhancer activity. Further, these enhancer-associated signals coincide with a candidate cis-regulatory element cataloged by the ENCODE consortium [43] (Fig. 2).

We also found evidence that IFNAR1.L1M2a shows inducible enhancer activity upon type I IFN signaling. ATAC signal increases markedly upon treatment of cells with interferon alpha (IFNɑ), and antisense PRO-Seq signal increases upon treatment with interferon beta (IFNβ) in GM12878 cells (Fig. 2B), in agreement with our hypothesis that bivalent LINEs may be primed for inducible regulatory activity.

We examined publicly available ChIP-seq data using the ReMap database [44] and identified many transcription factors which directly bind the predicted enhancer region across a variety of cell types (Additional file 5: Table S4). While many of these were associated with repression, we also found evidence for binding by STAT1, one of the transcription factors activated by type I IFN signaling through IFNAR1 and its partners. Orthogonally, we sought to identify transcription factor binding sites within the predicted enhancer region which might facilitate IFN inducibility. Using the Find Individual Motif Occurrences (FIMO) function from the MEME suite [45], we found a GAS motif within the predicted enhancer (Fig. 2A). GAS motifs are typically expected to respond to canonical type II IFN signaling pathways, non-canonical type I IFN signaling pathways, and other cytokines [34]. Together these findings strongly suggest that the IFNAR1.L1M2a element acts as an IFN-inducible enhancer element in B cells.

### CRISPR knockout of the IFNAR1.L1M2a putative enhancer element alters the local epigenetic landscape

**Fig. 3:**
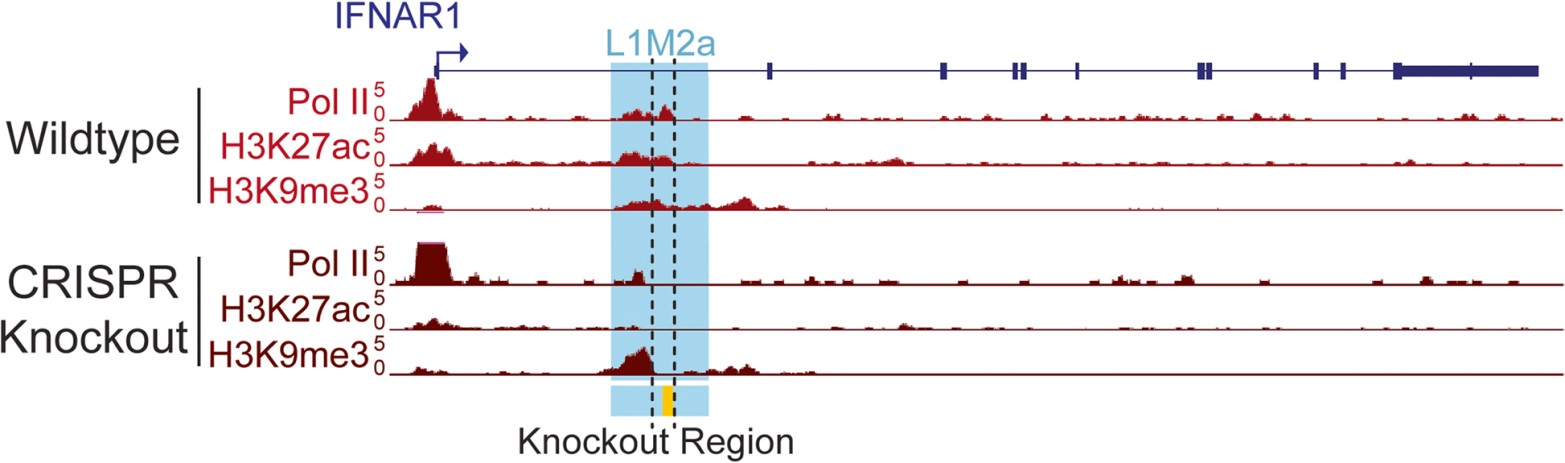
CRISPR knockout of IFNAR1.L1M2a.enh alters the local chromatin landscape. Wildtype GM12878 cells (light red), as well as a representative IFNAR1.L1M2a.enh knockout clone, L3.E4 (dark red) were characterized by CUT&Tag. The expected bivalent histone modifications and PolII occupancy are present in wildtype GM12878 cells, but both PolII and enhancer associated H3K27ac are lost, and upstream repressive H3K9me3 is increased in knockout clone L3.E4 cells

While the IFNAR1.L1M2a element shows epigenomic hallmarks of enhancer function, we sought to demonstrate this function empirically. We used CRISPR/Cas9 to test the regulatory activity of IFNAR1.L1M2a in the GM12878 cell line by deleting the internal ENCODE-predicted enhancer region and assessing the epigenetic and transcriptional consequences [46] (Additional file 6: Table S5). This target region encompassed the observed ATAC and PRO-Seq peaks, the center of the H3K27ac peak, and the GAS motif we hypothesized to be involved in IFN inducible enhancer activity. We generated four clonal cell lines with homozygous knockouts of the IFNAR1.L1M2a predicted enhancer sequence (IFNAR1.L1M2a.enh), and validated them by PCR and Sanger sequencing (Fig. 3, Additional file 1: Fig. S5, Additional file 6: Table S5).

We next assessed how the deletion affected the epigenetic landscape of the IFNAR1 locus by conducting CUT&Tag [47] on wild-type cells and one of the IFNAR1.L1M2a.enh knockout cells, profiling H3K27ac, H3K9me3, and RNA Polymerase II (PolII). This revealed a complete loss of H3K27ac signal across the whole IFNAR1.L1M2a locus surrounding the deleted region in the knockout cells (Fig. 3, Additional file 1: Fig. S6). We also observed an accumulation of H3K9me3 at the 5’ end of the IFNAR1.L1M2a element in cells where the internal enhancer region was removed (Fig. 3, Additional file 1: Fig. S6). PolII occupancy over the IFNAR1.L1M2a region was almost entirely lost in the knockout cell line (Fig. 3). Though reduced, some PolII signal remained in knockout cells in the upstream region of IFNAR1.L1M2a. However, it was no longer accompanied by the active enhancer mark H3K27ac. Interestingly, we also observed increased PolII signal at the IFNAR1 gene promoter in knockout cells. This may represent a decreased ability to initiate or elongate transcription, leading to the buildup of PolII occupancy at the transcription start site and depletion of PolII further downstream. This may be relevant to lost IFNAR1.L1M2a enhancer activity, or it may be the result of accumulated H3K9me3. Therefore, our CUT&Tag data established that deletion of the predicted enhancer within the IFNAR1.L1M2a element abolishes the markers of enhancer activity, and promotes buildup of repressive modifications.

### IFNAR1.L1M2a is an enhancer of IFNAR1 expression

**Fig. 4:**
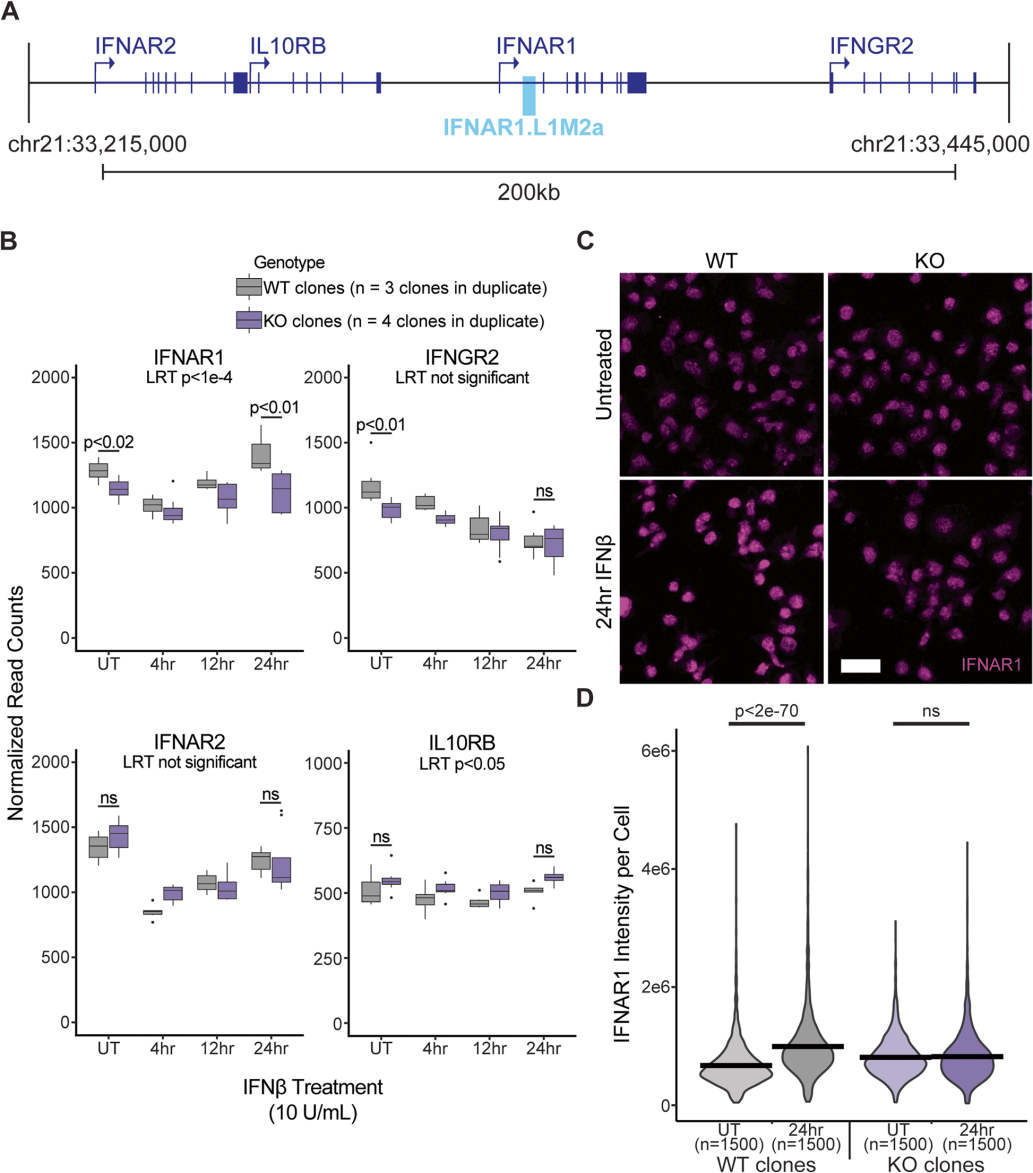
IFNAR1.L1M2a inducibly enhances transcription of IFNAR1 during type I IFN signaling. **A** IFNAR1 and three nearby genes, IFNAR2, IL10RB, and IFNGR2, were all considered to be possible targets of IFNAR1.L1M2a enhancer activity due to their proximity. **B** RNA sequencing of three wildtype clones (gray) and four IFNAR1.L1M2a.enh knockout clones (purple) for IFNAR1.L1M2a-proximal genes during a 24 h time course of IFNβ treatment. Pairwise differential expression analysis [48] at the untreated timepoint showed slightly but significantly lower baseline expression of IFNAR1 and IFNGR2 in knockout cells, and likelihood ratio tests [48] showed significant differences in response to IFNβ treatment of IFNAR1 and IL10RB. At the 24 h timepoint, IFNAR1 expression was slightly but significantly upregulated compared to the untreated timepoint in wildtype cells but not in knockout cells, and expression of IFNAR1 was significantly lower in knockout cells. **C**-**D** Immunofluorescence was used to confirm protein-level differences in IFNAR1 expression between wildtype cells (left, gray) and knockout cells (right, purple) in untreated (top, light) and IFNβ-treated (bottom, dark) conditions. In agreement with RNA-seq data, IFNAR1 expression was induced by IFNβ treatment in wildtype cells, but not in knockout cells. Scale bar indicates 25 microns

We next used RNA-seq to profile the transcriptional response of wildtype and knockout cells to type I IFN over a time course of 24 h (at 0 h, 4 h, 12 h, and 24 h). Because IFNAR1.L1M2a is nested within the first intron of IFNAR1, we predicted IFNAR1 to be the most likely regulatory target of IFNAR1.L1M2a. Three nearby genes were also considered as potential target genes based on their proximity to the element, with transcription start sites (TSS) within about 100 kb of the LINE: Interleukin 10 Receptor Subunit Beta (IL10RB), Interferon Alpha/Beta Receptor 2 (IFNAR2), and Interferon Gamma Receptor 2 (IFNGR2) (Fig. 4A). Differential expression analysis [48] of IFNAR1.L1M2a.enh knockout cells compared with wildtype cells showed a reduction in baseline expression of both IFNAR1 and IFNGR2, but no significant changes in the baseline expression of IFNAR2 or IL10RB (Fig. 4B, Additional file 7: Table S6). This is consistent with a TAD domain boundary evident in a published GM12878 Micro-C dataset [49] (Additional file 1: Fig. S4). In parallel, we used quantitative PCR (RT-qPCR) to assess IFNAR1 transcript levels and saw decreased levels of IFNAR1 in knockout compared to wildtype cells at all timepoints (Additional file 1: Fig. S7). Thus, our knockout experiment demonstrates that IFNAR2.L1M2a.enh acts as an enhancer of baseline IFNAR1 and IFNGR2 expression in the GM12878 cell line (Additional file 1: Fig. S8).

We also observed that transcription of IFNAR1 was upregulated by 24 h treatment with IFNβ in wildtype cells, and not in knockout cells. Correspondingly, we observed significantly lower levels of IFNAR1 RNA and protein in IFNβ-treated knockout cells compared with IFNβ-treated wildtype cells (Fig. 4B, C, Additional file 8: Table S7). This is consistent with the IFNAR1.L1M2a element acting as an inducible enhancer of IFNAR1. It should be noted, that while expression of IFNAR1 in knockout cells was significantly lower than in wildtype cells as observed by RT-qPCR, this assay did not capture the wildtype induced expression of IFNAR1 that we observed by RNA-Seq and immunofluorescence (Additional file 1: Fig. S7), likely due to differences in assay sensitivity.

To determine how the time course dynamics of the IFNβ-induced transcriptional response were affected by the IFNAR2.L1M2a.enh knockout, we used a likelihood ratio test (LRT) to compare gene expression profiles in wildtype and knockout cells over the 0, 4, 12 and 24 h treatment time course. Both IFNAR1 and IL10RB show significantly different expression profiles in knockout cells, but IFNAR2 and IFNGR2 do not (Fig. 4B). While IL10RB levels remained slightly elevated at all timepoints in the knockout cells, IFNAR1 levels were slightly reduced, and expression was not induced by prolonged IFNβ exposure as it was in their wildtype counterparts. These results support the hypothesis that IFNAR1.L1M2a includes an enhancer of IFNAR1 transcription which is active at steady state and is induced downstream of IFN signaling.

In wildtype cells the upregulated expression of IFNAR1 was not observed until 24 h after IFN exposure (Fig. 4B). This slow response suggests dependence on a pathway downstream of the initial type I IFN response, rather than a direct feedback loop. Based on the altered expression of IFNGR2 and the presences of a GAS motif within the IFNAR1.L1M2a.enh region, we considered an interaction between the initial type I IFN signal and a downstream activation of the type II IFN pathway, which might loop back and induce the upregulated expression of the IFNAR1 gene. However, qPCR showed no significant difference in the expression of IFNAR1 or of representative ISGs between wildtype and knockout cells in response to IFNɣ treatment (Additional file 1: Fig. S9). We also considered that knockout of IFNAR1.L1M2a may alter splicing of the IFNAR1 transcript that could complicate gene level differential expression analyses. However, un-guided transcript assembly revealed only one transcript isoform of IFNAR1 expressed in both wildtype and knockout cells, and no differential splicing between the two genotypes (Additional file 1: Fig. S8).

### Loss of IFNAR1.L1M2a.enh dampens responsiveness of cells to type I IFN signaling

**Fig. 5:**
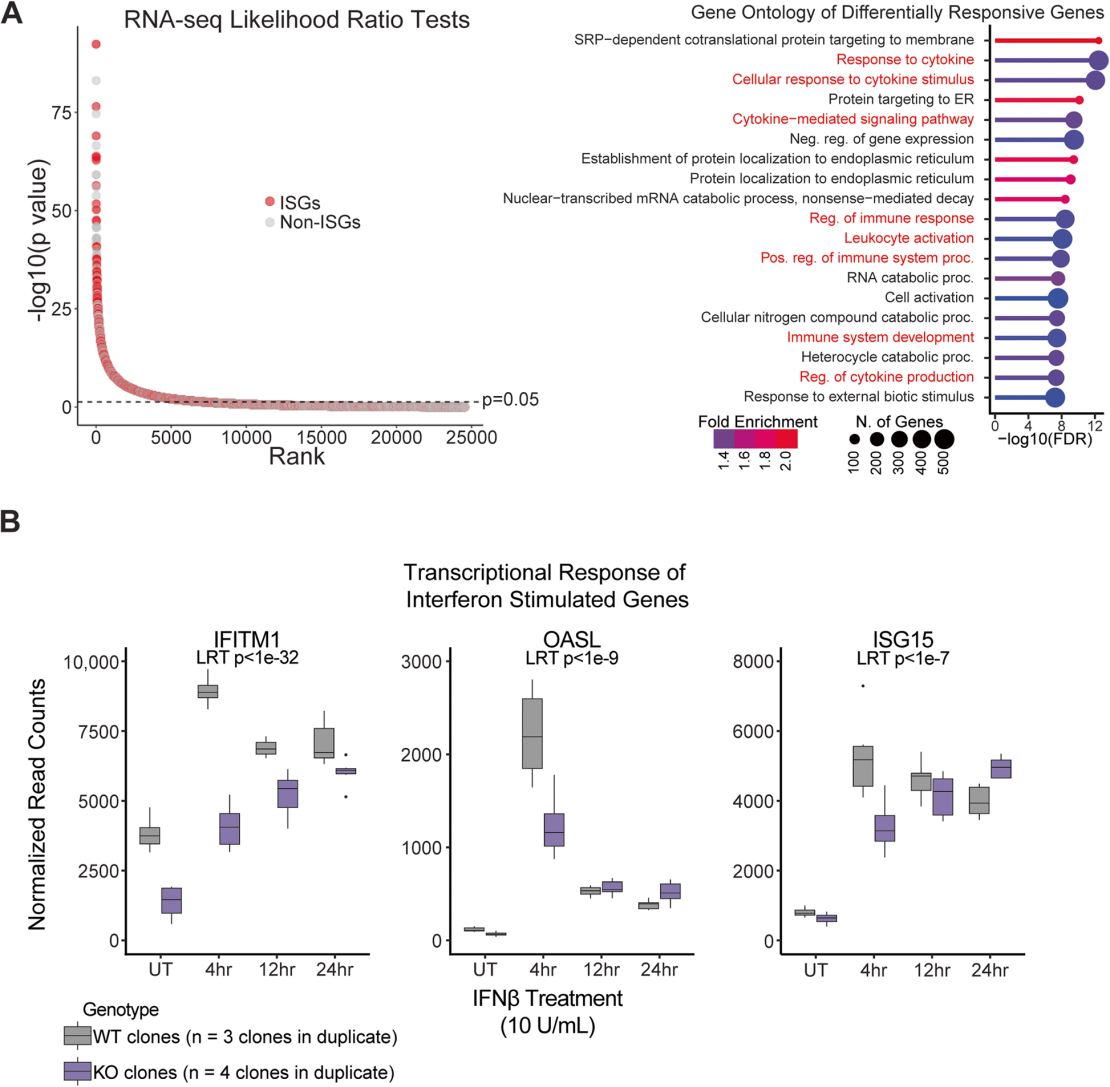
Deletion of IFNAR1.L1M2a.enh alters downstream transcriptional response to IFNβ. RNA sequencing of three wildtype (gray) and four IFNAR1.L1M2a.enh knockout clones (purple) during a 24 h time course of IFNβ treatment. **A** Likelihood Ratio Tests [48] of RNAsequencing data revealed many genes with significantly altered IFNβ responses in knockout cells compared to wildtype. Many of these genes were expected IFN stimulated genes (red) as identified by induction in wildtype cells at the 4 h timepoint [48] (Additional file 7: Table S6). Gene ontology [51] confirmed that significantly differentially responsive genes were enriched for immune-related biological processes (red). **B** RNA sequencing of representative expected ISGs showed a dampened initial induction in the knockout cells (purple) compared to wildtype cells (gray) and differential expression throughout the time course of IFNβ treatment according to likelihood ratio tests [48]

Since IFNAR1 receptor levels modulate cellular sensitivity to type I IFN signaling [50], we asked whether cells lacking IFNAR1.L1M2a.enh exhibit an altered overall IFN response based on gene expression dynamics. LRT tests on all genes over a 24 h time course of IFNβ treatment found that the transcriptional response of many genes was significantly altered in IFNAR1.L1M2a.enh knockout cells compared with wildtype cells [48] (Fig. 5A, Additional file 7: Table S6). Approximately 31% of the differentially responsive genes were ISGs, defined as genes significantly upregulated after 4 h of IFNβ treatment in the wildtype cells (Additional file 7: Table S6). In comparison, ISGs made up approximately 14% of those which were not significantly differentially responsive in the knockout cells. We also conducted gene ontology analysis on the differentially responsive genes and found that the significantly altered genes were enriched for immune-related pathways, prominently including cytokine responses [51] (Fig. 5A).

In IFNAR1.L1M2a.enh knockout cells, the initial transcriptional induction after 4 h of IFNβ exposure was significantly reduced in many canonical ISGs, including Interferon-Induced Transmembrane Protein 1 (IFITM1), 2'-5'-Oligoadenylate Synthetase Like (OASL), and Interferon Stimulated Gene 15 (ISG15) (Fig. 5B), indicating a dampened initial response to type I IFN signaling. These results were corroborated at the protein level by measuring IFITM1 abundance per cell by immunofluorescence. We observed that IFITM1 abundance was upregulated by IFN treatment in both wildtype and knockout cells, but to a significantly lower degree in knockout cells (Additional file 1: Fig. S10, Additional file 9: Table S8), consistent with reduced signal transduction. Together, these results indicate that IFNAR1-mediated signaling in response to type I IFN is regulated by IFNAR1.L1M2.enh in GM12878 cells.

## Discussion

Here, we showed how a LINE insertion exhibiting both repressive and enhancer-associated epigenetic markers can act as a cis-regulatory element. In the case example of the IFNAR1.L1M2a locus, multiple lines of epigenomic evidence indicated that an immune cell type-specific enhancer element existed within the LINE sequence. We confirmed that CRISPR knockout of the predicted enhancer in GM12878 B-lymphoblastoid cells both reduced baseline expression of the surrounding IFNAR1 gene and abolished its inducible upregulation by type I IFN signaling. We also observed that the downstream transcriptional response of the cells to type I IFN was significantly altered. Thus, we have characterized a lineage-specific LINE which functions as an inducible enhancer for a critical immune gene.

We speculate that IFNAR1.L1M2a has an important function in maintaining IFNAR1 levels by mediating a positive feedback loop driving IFNAR1 expression during type I IFN signaling. During signal transduction, activated IFNAR1/IFNAR2 complexes are actively internalized through clathrin-mediated endocytosis, and while IFNAR2 is recycled to the cell surface, IFNAR1 is thought to be degraded [52]. Therefore, an inducible feedback circuit to replenish IFNAR1 levels may serve to allow ongoing signaling. It is interesting to note that we observed an apparent decrease in expression of both IFNAR1 and its partner IFNAR2 at the 4 h timepoint of interferon signaling (Fig. 4B), that was then followed by induced expression. As this was seen in both wildtype and knockout cells, this phenomenon cannot be explained by IFNAR1.L1M2a.enh activity, but it may be of importance to the function of the interferon signaling pathway nevertheless. Downregulation of receptors has been observed previously during early timepoints of interferon exposure, but was attributed to the induced degradation of activated IFNAR1 rather than decreased expression of the receptor [53, 54]. The recovery of expression which we observed at later timepoints in wildtype, but not knockout cells, may implicate the IFNAR1.L1M2a enhancer in replenishing sensitivity to ongoing signaling.

Our study demonstrates that LINEs–including intronic LINEs marked by repressive chromatin marks–can exhibit cell type-specific enhancer activity. Previous studies have mostly considered LINEs to be strongly repressed in primary cells, only exhibiting gene regulatory activity when these repressive marks are aberrantly lost [55], but while much research on regulatory TEs has focused on ERVs [56], LINEs are now increasingly appreciated to play diverse transcriptional regulatory roles. LINEs can act as alternative promoters [57], alternative splice sites [58–60], and transcription factor binding sites [8, 61]. However, the biological significance of these elements has only recently been investigated using knockout experiments. One recent study experimentally demonstrated that a L1MC1 LINE acts as a regulatory boundary domain in GM12878 cells [62]. Another demonstrated that an L1MC5 element within the intron of IFNGR2 acts as a cryptic splice acceptor in T cells, resulting in transcriptional repression [63]. Finally, in mice, an Lx9 element was demonstrated to be an essential long non-coding RNA that inhibits the immune response [19]. Our functional characterization of IFNAR2.L1M2a.enh in immune cells provides a key demonstration that LINEs can also be co-opted as inducible enhancers. Even those elements that appear strongly repressed based on chromatin profiling data may maintain cell type-specific or context-dependent enhancer potential. This dichotomy may convey an inducible and/or heterogenous quality to these elements, representing a previously unstudied pool of transposon-derived, co-opted regulatory elements.

We note several limitations to our study. The observed differences in IFNAR1 expression between the wildtype and IFNAR1.L1M2a.enh knockout cells were significant, but the knockout did not result in total ablation of IFNAR1 expression or IFN signaling. It is likely that other regulatory elements in the vicinity play a role in regulating expression of this key gene. Moreover, while the transcriptional effects on IFNAR1 and the IFN response were consistent across multiple CRISPR knockout clones, it is possible that some of these changes may be due to inherent clonal variation due to the CRISPR editing process [64]. We attempted to mitigate this problem by comparing clonal knockout cell lines against wildtype cells that had been clonally expanded in the same way.

Another limitation is our use of GM12878 immortalized B-lymphoblastoid cells to study IFNAR1.L1M2a.enh, which we used due to their amenability to genome editing. While we observed the same patterns of overlapping repressive and active epigenetic markers in multiple primary immune cell types, without experimental confirmation of function, it is possible that the enhancer activity observed in the GM12878 line may differ in primary cells or in vivo.

Finally, the bivalent repressive and active epigenetic patterns that marked a subset of LINEs including IFNAR2.L1M2a were observed in bulk data. Our hypothesis that this pattern represents priming of inducible regulatory elements assumes that these overlapping repressive and enhancer-associated markers are truly bivalent, overlapping in individual cells. It is possible, however, that it indicates heterogeneity between cells in a bulk population rather than homogenous overlap. Our observations of IFNAR1.L1M2a supported our hypothesis of inducible enhancer activity, but the observation that steady state gene expression is also reduced may support the alternative, and by no means mutually exclusive hypothesis of heterogeneity.

## Conclusions

We have characterized a LINE-derived IFN-inducible enhancer of IFNAR1 gene expression: IFNAR1.L1M2a. We have shown that the deletion of this element not only alters the baseline transcription of the IFNAR1 gene, but also abolishes the IFN-induced upregulation of IFNAR1. These changes in expression lead to a dampening of the cellular type I IFN signaling response. This is among one of the first LINE-derived cis-regulators to be experimentally characterized, but there are likely many more LINEs serving important biological functions in human cells. The IFNAR1.L1M2a element characterized here was drawn from a set of LINEs exhibiting similar epigenetic patterns in human B cells, which may likewise possess inducible enhancer activities.

## Methods

### Transposon ChIP-Seq analysis

All analyses were conducted using the hg38 human genome assembly. ChIP-Seq datasets for primary blood cells were downloaded from the Blueprint Database [28], as pre-processed bed and bigwig files. ChIP-Seq datasets for GM12878 lymphoblastoid cells were downloaded from the ENCODE [29] as raw data and processed as below in “ChIP-Seq Data Analysis”. Enrichment of annotated transposon families within ChIP-Seq peak calls was assessed using Giggle [38] and the hg38 DFAM 2.0 Repeatmasker annotation [65, 66]. Giggle-derived enrichment scores were visualized as heatmaps using seaborn.clustermap [67]. Only families with giggle enrichment scores above 100 or below -100 are displayed in Fig. 1. Full heatmaps are found in additional files. Annotated LINEs were filtered for a minimum length of 500 base pairs to remove fragments. Filtered LINEs were compared against ChIP-Seq datasets for H3K4me1, H3K27ac, and H3K9me3 using the BEDTools utilities Intersect, Sort, and Merge [68]. LINEs overlapping either H3K4me1 or H3K27ac were designated as enhancer-like, and LINEs overlapping H3K9me3 were designated as repressed. LINEs in both the enhancer-like and repressed lists were designated as bivalent. Heatmaps and metaplots of ChIP-Seq signal at each LINE on the compiled lists were visualized using deepTools functions computeMatrix and plotHeatmap [69]. Commands used are available at https://github.com/crmnbttlr/IFNAR1.L1M2a_2023.

### Motif Identification

The MEME tool Find Individual Motif Occurrences v5.5.2 (FIMO) [45] was used to identify STAT1 binding motifs, including the GAS motifs MA0137.2 and MA0137.3 from the JASPAR CORE [70]. The FIMO search was not strand specific, and applied a *p*-value threshold of 0.0004.

### ATAC-Seq library preparation

Cells were treated with either 100 U/ml human IFNα2 (#HZ-1066, ProteinTech) or 0.001% DMSO for 1 h. Libraries were prepared using a protocol modified from previously published protocols [71]. Cells were pelleted at 4 °C and supernatant aspirated. Cells were resuspended in 50ul chilled lysis buffer (10 mM Tris–HCl pH7.4, 10 mM NaCl, 3 mM MgCl_2_, 0.1% IGEPAL, 0.1% Tween-20, 0.01% Digitonin), and incubated for 5 min on ice, then 1 ml of wash buffer (10 mM Tris–HCl pH7.4, 10 mM NaCl, 3 mM MgCl_2_, 0.1% Tween-20) was added and mixed. Cells were then pelleted again for 10 min at 4 °C and the supernatant aspirated. Pellets were resuspended in 50ul of transposition buffer (25ul Tagmentation DNA Buffer Illumina #15,027,866, 2.5 ul Tagment DNA Enzyme 1 Illumina #15,027,865, 0.5ul Digitonin diluted 1:1 in water, 0.5ul 10% Tween-20, 5ul water, and 16.5ul PBS), then incubated for 30 min at 37 °C. Samples were purified using the Zymo DNA Clean and Concentrator Kit (#D4014, Zymo Research). Fragments were amplified with a 5 cycle PCR, then an additional cycle with Nextera DNA CD Indices (#20,015,882, Illumina), then re-purified with the DNA Clean and Concentrator Kit. Fragments were filtered using a BluePippin (Sage Science) for fragments smaller than 100 base pairs. Libraries were quantified using Qubit (Invitrogen) and pooled, then bead-cleaned using 1.5 volumes of AMPure XP beads (#A63881, Beckman Coulter, and eluted in EB buffer (#19,086, Qiagen). Libraries were sequenced using a NextSeq 500 at a depth of ~ 20 million reads per sample.

### ATAC-Seq data analysis

Raw datasets were processed through the following pipeline. Raw fastq files were quality checked using FastQC v0.11.5 [72]. Adapters were trimmed using BBDuk v38.05 [73], and reads were aligned using HISAT2 v2.1.0 [74] with options –new-summary –very-sensitive –no-spliced-alignment. Unmapped reads were filtered using Samtools v1.8 [75] with option -F 4. Read duplicates were removed using Sambamba v1.8 markdup with options –remove -duplicates, –overflow-list-size = 300000 [76]. Bedgraph files were then produced using deepTools v3.0.1 [69] bamCoverage with options –binSize 1, –normalizeUsing CPM. Data was visualized by generating bigwig files using BedGraphToBigWig [77], which were uploaded to the University of California Santa Cruz genome browser [78].

### Cell culture conditions

GM12878 lymphoblastoid cells were obtained from the Coreill Institute (Camden, NJ). Cells were cultured in Roswell Park Memorial Institute (RPMI) media (#72,400–047, Gibco) with 15% Fetal Bovine Serum (#S11150, R&D Systems) by volume and 1% Penicillin/Streptomycin (#15,140–122, Gibco), according to the recommendations of the Coriell Institure and were grown at 37 °C in 5% CO_2_.

### CRISPR design and cell line generation

Guides for CRISPR/Cas9 knockout of IFNAR1.L1M2a.enh were designed using the UCSC genome browser track “CRISPR/Cas9-NGG Targets, whole genome [79].” Three guides were chosen with low off-target probability and high efficiency scores, with one upstream and two downstream of the IFNAR1.L1M2a.enh region. Guide sequences are listed in Additional file 6: Table 5.

CRISPR/Cas9 knockout was performed using IDT’s Alt-R system and the Neon Transfection System (#MPK5000, Thermo Fisher). Guide RNAs were purchased from IDT and combined with Alt-R tracrRNA (#1,072,533, IDT) in nuclease free IDTE (#11–01-02–02, IDT), heated to 95 °C for 5 min and then cooled to room temperature. Alt-R S.P. HiFi Cas9 Nuclease V3 (#1,081,060, IDT) was diluted in Neon Resuspension Buffer R (#MPK1096, Thermo Fisher) and added to the RNA mixture for, incubated 10–20 min at room temperature. Alt-R Cas9 Electroporation Enhancer (#1,075,916, IDT) was added. Electroporation was performed according to Neon Transfection System recommended protocols, and GM12878 cells were electroporated using three 10 ms pulses at 1200 V.

Single cell dilutions were plated to 92-well plates in order to isolate clonal cell lines. Once expanded, clonal lines were screened for homozygous knockout of IFNAR1.L1M2a.enh. Genomic DNA was isolated from cells using Quick Extract DNA Extraction Solution (#QE09050, Lucigen). Initial screening for knockouts was performed by PCR amplification of the locus. Primer sequences are listed in Additional file 6: Table 5. Clones with apparent homozygous knockouts were then confirmed and characterized by Sanger sequencing using the CloneJET PCR Cloning Kit (#K1232, Thermo Fisher).

### CUT&Tag for ChIP-Seq library preparation

Cells were either untreated or cultured in media containing 10 U/ml human IFNβ (#HZ-1298, ProteinTech) for 4 h. CUT&Tag was performed using a protocol modified from Steven Henikoff’s lab [47]. Briefly, nuclei were isolated from approximately 500,000 cells, then bound to Concavalin A beads (#BP531, Bangs Laboratories). Primary rabbit antibodies against Phospho-Rpb1 CTD Ser5 D9N5I (#13,523, Cell Signaling Technology), H3 acetyl K27 (#ab4729, Abcam), H3 tri-methyl K9 (#ab8898, Abcam), and mouse IgG (#ab46540, Abcam) were introduced and incubated overnight. Nuclei were washed and a guinea pig anti-rabbit secondary antibody (#ABIN101961, Antibodies-Online) was introduced the next day and incubated overnight. pAG-Tn5 (#15–1017, EpiCypher) was used at a 1:40 dilution. Tagmentation and Chloroform DNA extraction were followed by 14 cycles of PCR amplification using barcoded Nextera adapter primers. Quantification and quality control were performed using Qubit (Invitrogen) and TapeStation 4200 (Agilent). Barcoded samples were pooled and sequenced at a depth of ~ 5 million reads per sample by the University of Colorado Anschutz Cancer Center Genomics Core using NovaSEQ 6000.

### ChIP-Seq data analysis

Raw sequencing datasets were processed through the following pipeline. Raw fastq files were quality checked using FastQC v0.11.8 [72], adapter sequences were trimmed using BBDuk v38.05 [73], and reads were aligned using BWA-MEM v0.7.15 [80]. Reads were filtered for alignment quality using samtools v1.10 [75]. Peaks were then called using MACS2 v2.1.1 [77]. Data was visualized by generating bigwig files using BedGraphToBigWig [77], which were uploaded to the University of California Santa Cruz genome browser [78]. Scripts and variables for these programs are available at https://github.com/crmnbttlr/IFNAR1.L1M2a_2023.

### RNA-Seq library preparation

Cells were either untreated or were cultured in media containing 10 U/ml human IFNβ (#HZ-1298, ProteinTech) for either 4, 12, or 24 h. Wildtype GM12878 cells, clonal wildtype cells (clones 2D3, 2D9, and 2F3) and clonal knockout cells (clones L3.A4, L3.E4, MN6.G11, and MN8.C12) were each collected in duplicate for each of the four treatment conditions. RNA was extracted from cells using the Zymo Quick RNA Miniprep Plus Kit (#R1504, Zymo Research) according to manufacturer protocols. RNA-Seq libraries were prepared using the KAPA mRNA Capture Kit (#KK8541, Roche), using their recommended protocol. Libraries were sequenced at a depth of ~ 15 million reads per sample by the University of Colorado Anschutz Cancer Center Genomic Core using NovaSeq6000.

### RNA-Seq data analysis

Raw RNA-Seq datasets were processed through the following pipeline. Files were quality checked using FastQC v0.11.8 [72], reads were aligned using HISAT2 v2.1.0 [74], and uniquely assigned were retained using samtools v1.10 with a filter of MAPQ >  = 10 [75]. Reads were assigned to annotated genes using featureCounts v1.6.2 [81]. RNA-Seq libraries were collected in two batches, so batch correction was performed using ComBat-seq [82] with 12 groups of clones at treatment timepoints. Differential expression was assessed between treatment conditions, clones, and genotypes using DESeq2 v1.26.0 [48]. DESeq2 was also used to conduct likelihood ratio tests (LRT) using a design of “ ~ genotype + time + genotype:time” and a reduced model of “ ~ time”. Scripts and variables for these programs are available at https://github.com/crmnbttlr/IFNAR1.L1M2a_2023.

### Spinning disc confocal microscopy

Cells were either untreated or cultured in media containing 10 U/ml human IFNβ (#HZ-1298, ProteinTech) for 24 h. Cells were then pelleted by centrifugation at 500 rpm for 5 min. Media was aspirated. Cells were resuspended and spun down again, twice in serum-free RPMI media, then added dropwise to clean, sterile glass coverslips treated with human fibronectin (#FC010, EMD Millipore) to allow them to settle onto the glass. Media was aspirated, cells were washed once with phosphate buffered saline (PBS), then fixed by treating for 25 min with 4% paraformaldehyde (#15,710, VWR). Coverslips were washed twice with Hank’s Balanced Salt Solution (HBSS), then stained with 5 ug/ml Wheat Germ Agglutinin Alexa Fluor 488 (WGA-AF488, #W11261, Thermo) for 10 min at room temperature, protected from light. Coverslips were then washed once with PBS, then cells were permeabilized by treatment with 0.2% Triton × 100 (#T8787, Sigma-Aldrich) for 10 min at room temperature. Coverslips were washed again with PBS, then stained with DAPI (#D9542, Sigma-Aldrich), diluted 1:1000 in PBS, for 5 min at room temperature. They were washed again with PBS, then blocked for 30 min in a solution of 10% bovine serum albumin (BSA, #A9418, Sigma-Aldrich), dissolved in PBS. Primary antibody staining was conducted for 1 h at room temperature. IFNAR1 was labeled using a rabbit anti-IFN-alpha/beta R1 antibody (#NBP2-67,339, Novus Biologicals). IFITM1 was labeled using a rabbit anti-IFITM1 Alexa Fluor 488 antibody (#NBP2-89230AF488, Novus Biologicals). Both were diluted 1:200 in a solution of 6% BSA in PBS. After primary staining, coverslips were washed with PBS, then stained with a secondary antibody, a goat anti-Rabbit IgG H&L Alexa Fluor 647 (#ab150079, Abcam), diluted 1:200 in a solution of 6% BSA in PBS for 1 h at room temperature. Coverslips were then washed three times for 10 min in a wash solution of 0.2% BSA and 0.05% Triton × 100 in PBS. Coverslips were then mounted to glass slides in a mounting medium of Fluoromount G (#0100–01, SouthernBiotech), and sealed around the edges with clear nail polish. Imaging was performed using a Nikon Ti-E Spinning Disc Confocal Microscope, with 488 nm (20%), 405 nm (20%) and 640 nm (25%) lasers, a 40 × air objective (Nikon), and an EMCCD camera (Andor iXon Ultra 888) at 300 ms exposure and 10 MHz EM Gain.

### Image analysis

Immunofluorescence images were analyzed using ImageJ [83] to mask and segment single cells and measure fluorescence intensity per cell in the 647 nm channel. Masking and segmenting was done using the WGA and DAPI channels. Three-channel images were opened in ImageJ as hyperstacks. A Gaussian blur was applied to the DAPI channel using a radius of 2. Thresholding was manually applied to maximized nucleus coverage and minimize background noise, and the mask was converted to a region of interest (ROI). A de-speckle function was applied to the WGA channel and thresholding was applied manually. The ROI derived from the DAPI channel was then filled to create a composite mask from the DAPI and WGA channels. An “open” transform was applied to remove remaining noise. Holes were filled if necessary. A watershed function was used to delineate single cells. ROIs for each cell were generated using the “analyze particles” function, with a minimum area of 30 microns^2^, with circularity between 0 and 1, excluding edges and including holes. Generated ROIs were manually inspected, and incorrectly segmented ROIs that either included multiple cells or partial cells were removed from the ROI manager. Measurements of area, mean, standard deviation, and integrated density were collected for all ROIs remaining in the manager. Integrated density per cell was used to approximate protein abundance. A pipeline in ImageJ is available at https://github.com/crmnbttlr/IFNAR1.L1M2a_2023.

### Gene expression analysis by RT-qPCR

Cells were either untreated or were cultured in media containing 1000 U/ml human IFNɣ (#485-MI, R&D Systems) for 4 h before RNA extraction. Duplicates of two wildtype clones (clones 2D3 and 2D9) and two knockout clones (clones L3.A4 and MN8.C12) were treated. RNA was extracted using the Zymo Quick RNA Miniprep Plus Kit (#R1504, Zymo Research) according to manufacturer protocols. RT-qPCR was performed in a 384-well plate using the Luna Universal One-Step RT-qPCR Kit (#E3005S, New England Biolabs) on a Bio-Rad CFX Opus 384 Real-Time PCR System. Normalized expression was calculated as 2 to the power of the difference between the Cq value of the gene of interest and the Cq value of CTCF. CTCF was quantified using primers ACCTGTTCCTGTGACTGTACC and ATGGGTTCACTTTCCGCAAGG. IFNAR1 was quantified using primers CGCCTGTGATCCAGGATTATCC and TGGTGTGTGCTCTGGCTTTCAC. IFITM1 was quantified using primers GGCTTCATAGCATTCGCCTACTC and AGATGTTCAGGCACTTGGCGGT.

### Transcript isoform analysis

RNA-Seq from all wildtype vs all knockout cells was used as input for Stringtie [84] to assemble transcripts without a reference guide and assess any differential splicing, with settings -j 5 and -f 0.25. Stingtie –merge was then used to compile all wildtype datasets and all knockout datasets.

### Supplementary Information

**Additional file 1: Supplemental Figure 1.** Enrichment of histone marks among transposon families. Giggle [38] was used to score enrichment (red) or depletion (blue) of transposon families overlapping the repressive histone modification H3K9me3 (top) and active histone modification H3K27ac (bottom). (A) Long Interspersed Nuclear Element (LINE) families. (B) Endogenous Retrovirus (ERV) families. (C) Short Interspersed Nuclear Element (SINE) families. (D) DNA transposon families. **Supplemental Figure 2.** Overlap of repressive and active histone marks at LINEs in naïve B cells. Heatmaps and metaplots of individual LINE-1s (A) and LINE-2s (B) that overlap repressive H3K9me3 signal only (navy), active marks H3K4me1 or H3K27ac (yellow), both H3K9me3 and active marks (cyan), or which overlap none of these three histone marks (green). **Supplemental Figure 3.** Gene function enrichment near epigenetically marked LINE-1s in naïve B cells. GREAT [85, 86] was used to assess gene ontology of likely target genes near LINE-1s marked by repressive H3K9me3 only (A), bivalent LINE-1s (B), and LINE-1s with only the enhancer-associated marks (C). The top 20 terms are shown, and their p values are plotted. Both enhancer-like and bivalent LINE-1s, but not repressed LINE-1s were enriched near genes involved in immune cell functions in these naïve B cells. **Supplemental Figure 4.** The IFNAR1 gene locus. (A) The IFNAR1.L1M2a element (light blue) lies within the first intron of the IFNAR1 gene, and includes a predicted distal enhancer [43, 65] (B) Evolutionary conservation of the IFNAR1.L1M2a element [42]. (C) Transcription factor binding peaks at the predicted enhancer [44]. (D) ChIP-seq data from primary human monocytes and macrophages [28] shows that not all immune cell types exhibit bivalent epigenetics at this locus. (E) Liu et al collected ChIP-seq data in their study [14] which shows that H3K9me3 overlap with the L1M2a element is dependent on the function of the HUSH complex, including the MORC2 and TASOR proteins. **Supplemental Figure 5.** Sequencing validation of CRISPR knockout of IFNAR1.L1M2a.enh. Four clonal lymphoblastoid cell lines (L3.A4, L3.E4, MN6.G11, and MN8.C12) were isolated with homozygous CRISPR knockout of IFNAR1.L1M2a.enh. The knockout regions were validated by sanger sequencing, and the alleles are displayed here, with the deleted regions marked with dotted lines. **Supplemental Figure 6.** Chromatin profiling of the IFNAR1.L1M2a locus upon IFNβ signaling. CUT&Tag [47] was used to collect ChIP-seq of active H3K27ac (top) and repressive H3K9me3 (bottom) in wild type (light red) and knockout (dark red) lymphoblastoid cells in untreated conditions and upon treatment with IFNβ for 4 hours. **Supplemental Figure 7.** Quantification of IFNAR1 expression by RT-qPCR. RT-qPCR was used in parallel with RNA-Seq to assess changes in the transcription of IFNAR1 upon IFNAR1.L1M2a.enh knockout. Significantly lower expression of IFNAR1 was observed in knockout cells (purple) compared with wildtype (gray) at all timepoints, using a student’s t-test. * indicates p<0.05. ** indicates p<0.01. *** indicates p<0.001. **Supplemental Figure 8.** RNA-Seq and transcript assembly at the IFNAR1 locus. (A) Transcript assembly using Stringtie [84] shows no differential splicing between wildtype and knockout cells at the IFNAR1 gene. (B) Buildup of RNA-Seq reads across the IFNAR1 gene in wildtype (light blue) and knockout (dark blue) cells from representative datasets (wildtype clone 2D3 and knockout clone L3.A4). **Supplemental Figure 9.** Transcriptional Response of wildtype and knockout cells to IFNɣ. Quantitative PCR measuring expression, normalized to CTCF, of IFNAR1 (A) and representative IFN stimulated gene IFITM1 (B), in wildtype compared with IFNAR1.L1M2a.enh knockout cells, under untreated and IFNɣ treated conditions. Two clonal cell lines of each genotype were used, each in duplicate. There was no significant difference between wildtype and knockout cells under the same treatment conditions. **Supplemental Figure 10.** Immunofluorescence of IFITM1. Immunofluorescence was used to approximate quantify IFITM1 protein abundance at the single cell level. (A) Representative images of IFITM1 labeling in wildtype and IFNAR1.L1M2a.enh knockout cells, under untreated and IFNβ-treated conditions. Scale bar is 25 microns. (B) Quantification of IFITM1 staining intensity per cell. Both wildtype and knockout cells show induction of IFITM1, though in knockout cells the induction appears less robust, in agreement with RNA-seq data (Fig. 5).

**Additional file 2: Supplemental Table 1.** Epigenetically Marked LINE-1s in Primary Naive B Cells. Lists of LINE-1s in the human genome which overlap the epigenetic marks of interest in naive B cells [28].

**Additional file 3: Supplemental Table 2.** Epigenetically Marked LINE-1s in Lymphoblastoid GM12878 Cell Line. Lists of LINE-1s in the human genome which overlap the epigenetic marks of interest in lymphoblastoid GM12878 cells [29].

**Additional file 4: Supplemental Table 3.** Genes Proximal to Epigenetically Marked Genes in Primary Naive B Cells. Lists of genes with potential cis interaction with epigenetically marked LINE-1s in naive B cells [28], according to GREAT analysis [85, 86].

**Additional file 5: Supplemental Table 4.** Transcription Factor Binding in the IFNAR1.L1M2a.enh region according to ReMap. List of transcription factors found to bind within the IFNAR1.L1M2a.enh region in various cell types, according to ReMap 2022 [44].

**Additional file 6: Supplemental Table 5.** CRISPR Guides and Primers. Sequences of guides used to conduct CRISPR/Cas9 knockout of IFNAR1.L1M2a.enh, PCR primers used to validate knockouts, and sequences of knockout regions in four clonal knockout cell lines: L3.A4, L3.E4, MN6.G11, and MN8.C12.

**Additional file 7: Supplemental Table 6.** DESeq Differential Expression Analysis Outputs. Tabular outputs of differential sequencing analysis using DESeq2 [48], including comparisons of clonal wildtype cell lines 2D3, 2D9, and 2F3 against clonal knockout cell lines L3.A4, L3.E4, MN6.G11, and MN8.C12. Likelihood ratio test analysis across a timecourse of 0hr, 4hr, 12hr, and 12 hr IFNβ treatment is also included, as is comparison of 0hr against 4hr IFNβ treatment in the wildtype clonal cell lines. Normalized counts are also listed for non-clonal wildtype cells, but were not included in the differential analysis.

**Additional file 8: Supplemental Table 7.** Immunofluorescence of IFNAR1. Tables of IFNAR1 immunofluorescence intensity, as well as area-normalized mean intensity, for segmented individual cells. Datasets were randomly subset to normalize *n* values.

**Additional file 9: Supplemental Table 8.** Immunofluorescence of IFITM1. Tables of IFITM1 immunofluorescence intensity, as well as area-normalized mean intensity, for segmented individual cells. Datasets were randomly subset to normalize *n* values.

## Data Availability

RNA-Seq and CUT&Tag datasets generated during this study are available at the NCBI Gene Expression Omnibus (GEO) through accession number GSE225826, and ATAC-Seq datasets are available through accession number GSE217032. PRO-Seq datasets used in this study are available at accession number GSE217294 (GSM6716757, GSM6716758, GSM6716769 and GSM6716770) and ChIP-Seq datasets reanalyzed in this study are available from the Blueprint Database and Cistrome Database. Genomic, epigenomic, and transcriptomic datasets can be visualized through a UCSC Genome Browser session at https://genome.ucsc.edu/s/cabu5244/IFNAR1.L1M2a%20CRISPR%20KO. Scripts used for computational analyses and image analysis are available at https://github.com/crmnbttlr/IFNAR1.L1M2a_2023.
