# Supplementary material for: An intronic LINE-1 regulates IFNAR1 expression in human immune cells
Source: Mob DNA. 2023 Nov 30;14:20. doi: 10.1186/s13100-023-00308-3 (PMC10688052; doi:10.1186/s13100-023-00308-3)
Supplement: Supplementary file 1 — Additional file 1: Supplemental Figure 1. Enrichment of histone marks among transposon families. Giggle [38] was used to score enrichment (red) or depletion (blue) of transposon families overlapping the repressive histone modification H3K9me3 (top) and active histone modification H3K27ac (bottom). (A) Long Interspersed Nuclear Element (LINE) families. (B) Endogenous Retrovirus (ERV) families. (C) Short Interspersed Nuclear Element (SINE) families. (D) DNA transposon families. Supplemental Figure 2. Overlap of repressive and active histone marks at LINEs in naïve B cells. Heatmaps and metaplots of individual LINE-1s (A) and LINE-2s (B) that overlap repressive H3K9me3 signal only (navy), active marks H3K4me1 or H3K27ac (yellow), both H3K9me3 and active marks (cyan), or which overlap none of these three histone marks (green). Supplemental Figure 3. Gene function enrichment near epigenetically marked LINE-1s in naïve B cells. GREAT [85, 86] was used to assess gene ontology of likely target genes near LINE-1s marked by repressive H3K9me3 only (A), bivalent LINE-1s (B), and LINE-1s with only the enhancer-associated marks (C). The top 20 terms are shown, and their p values are plotted. Both enhancer-like and bivalent LINE-1s, but not repressed LINE-1s were enriched near genes involved in immune cell functions in these naïve B cells. Supplemental Figure 4. The IFNAR1 gene locus. (A) The IFNAR1.L1M2a element (light blue) lies within the first intron of the IFNAR1 gene, and includes a predicted distal enhancer [43, 65] (B) Evolutionary conservation of the IFNAR1.L1M2a element [42]. (C) Transcription factor binding peaks at the predicted enhancer [44]. (D) ChIP-seq data from primary human monocytes and macrophages [28] shows that not all immune cell types exhibit bivalent epigenetics at this locus. (E) Liu et al collected ChIP-seq data in their study [14] which shows that H3K9me3 overlap with the L1M2a element is dependent on the function of the HUSH complex, including the MOR [file 13100_2023_308_MOESM1_ESM.docx]

**Additional File 1. Supplemental Figures**

**
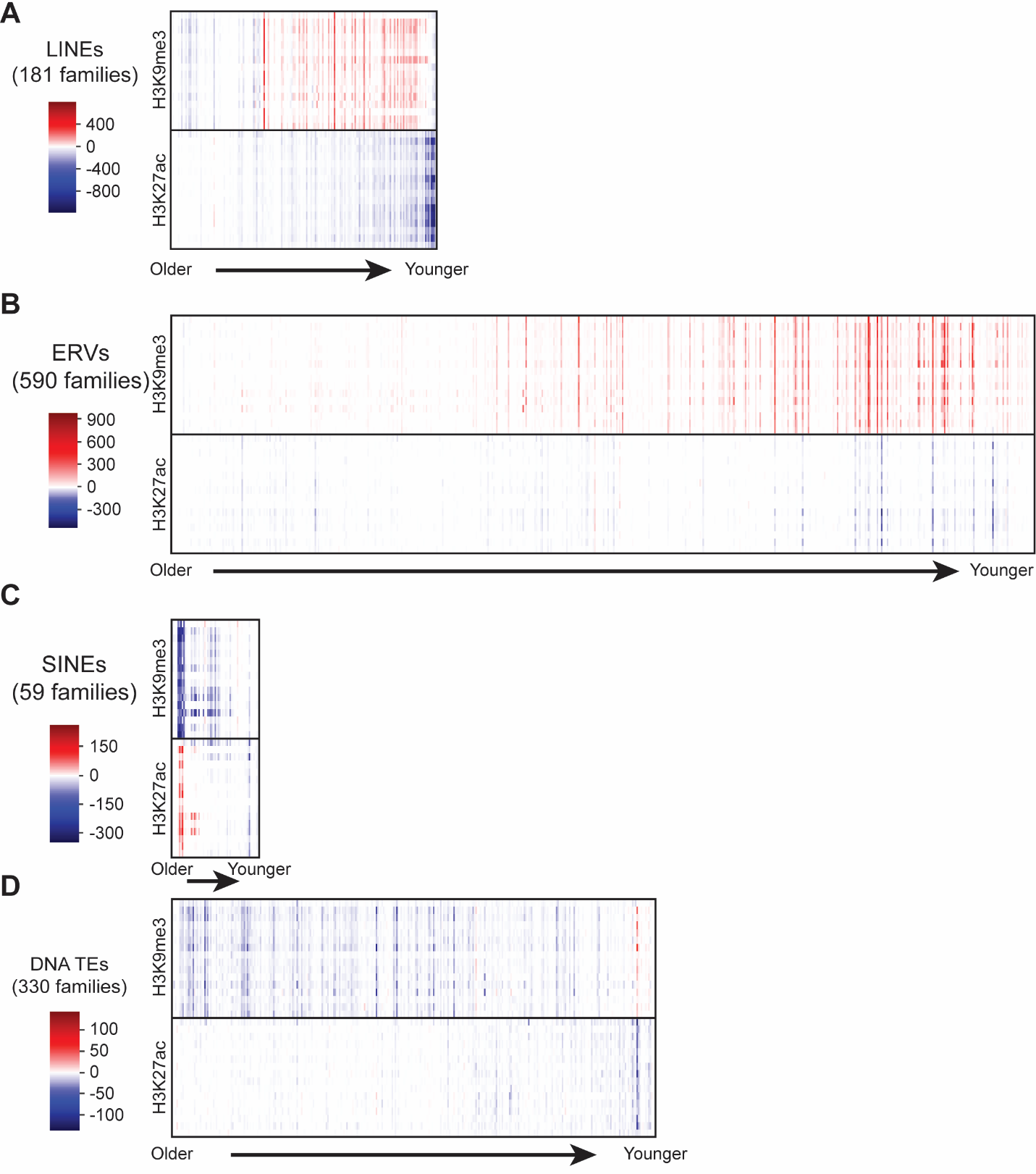
 Supplemental Figure 1. Enrichment of histone marks among transposon families.** Giggle[64] was used to score enrichment (red) or depletion (blue) of transposon families overlapping the repressive histone modification H3K9me3 (top) and active histone modification H3K27ac (bottom). **(A)** Long Interspersed Nuclear Element (LINE) families. **(B)** Endogenous Retrovirus (ERV) families. **(C)** Short Interspersed Nuclear Element (SINE) families. **(D)** DNA transposon families.

**
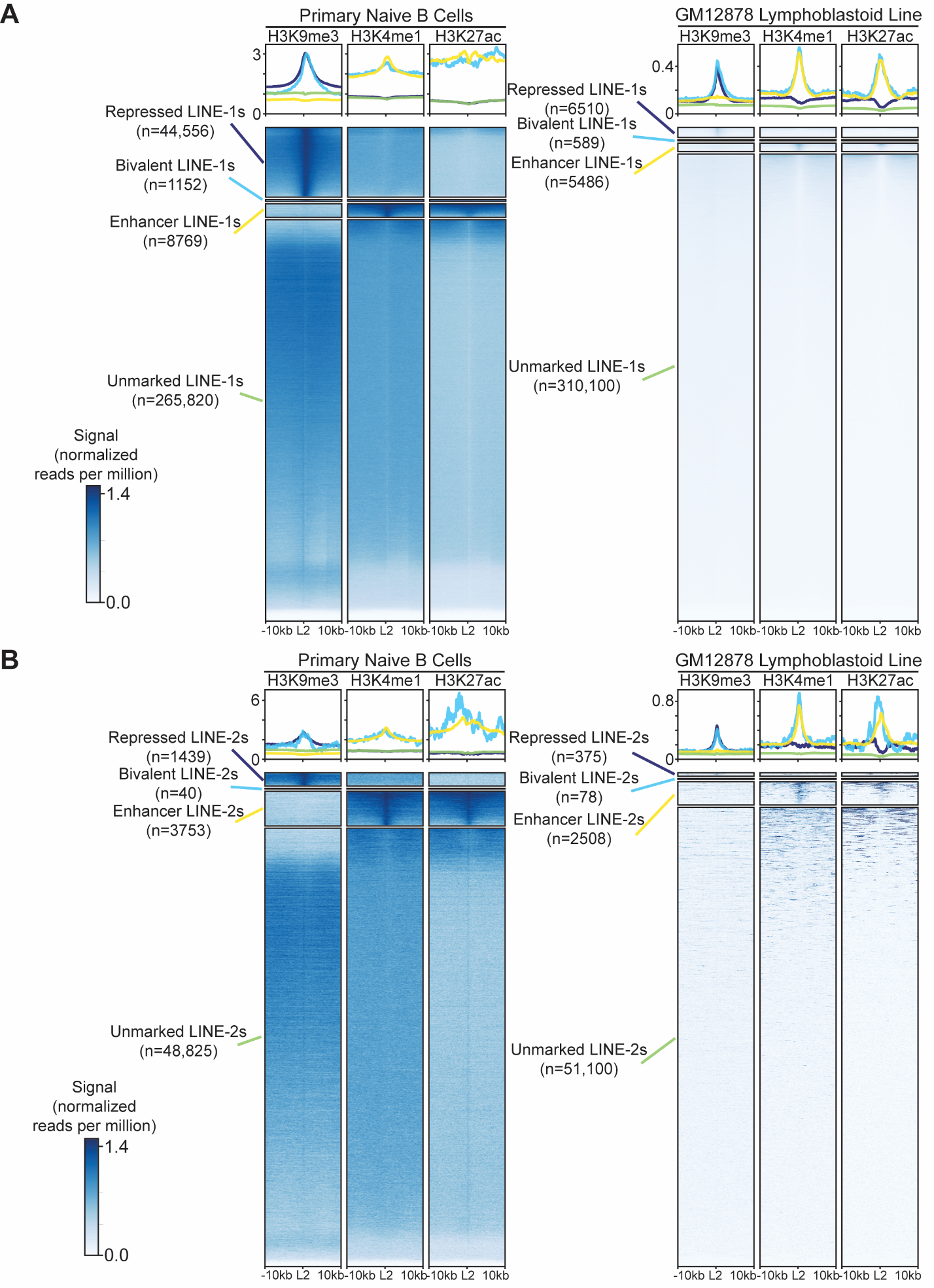
 Supplemental Figure 2. Overlap of repressive and active histone marks at LINEs in naïve B cells.** Heatmaps and metaplots of individual LINE-1s **(A)** and LINE-2s **(B)** that overlap repressive H3K9me3 signal only (navy), active marks H3K4me1 or H3K27ac (yellow), both H3K9me3 and active marks (cyan), or which overlap none of these three histone marks (green).

**
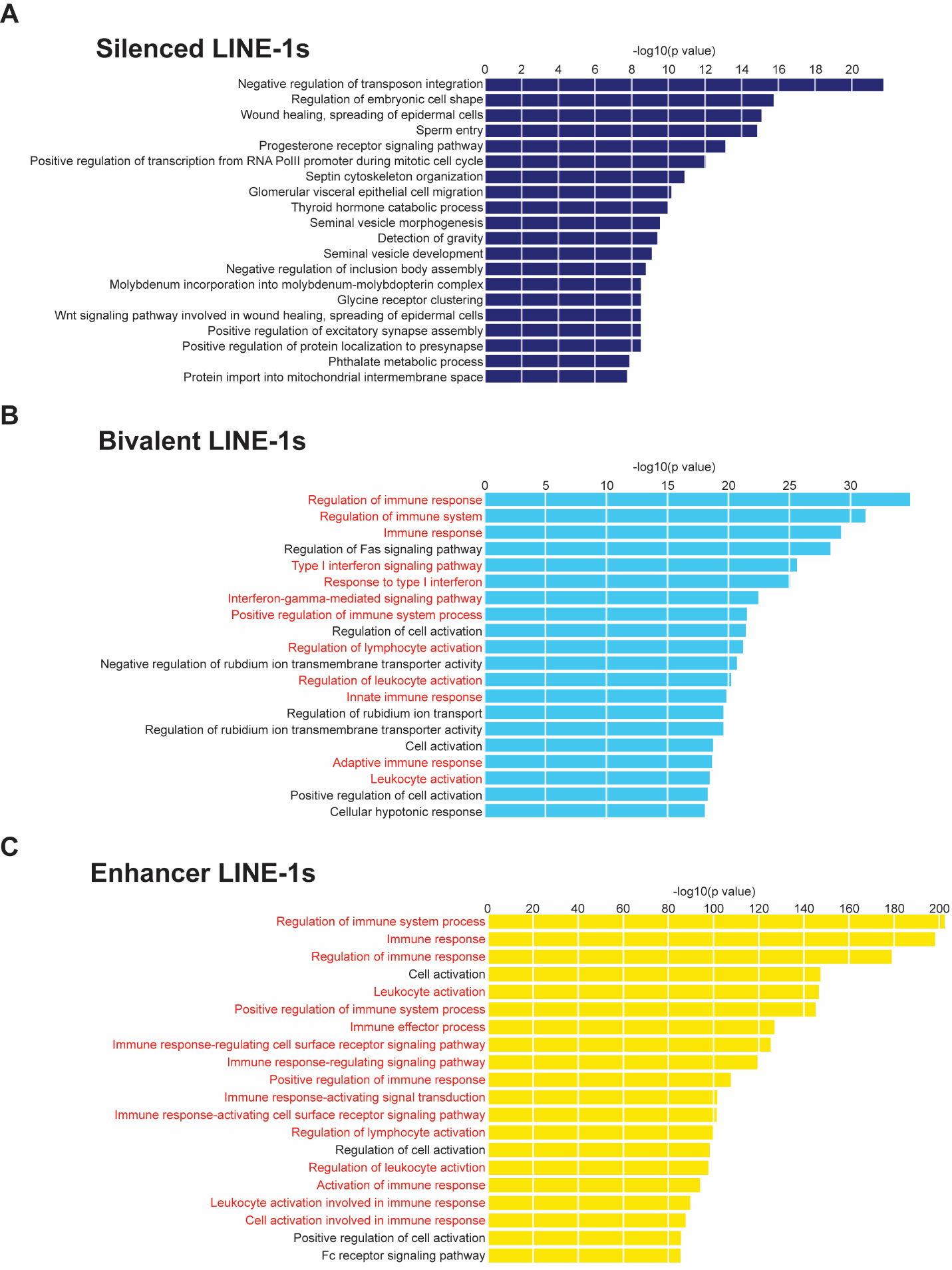
**

**Supplemental Figure 3. Gene function enrichment near epigenetically marked LINE-1s in naïve B cells.** GREAT[85,86] was used to assess gene ontology of likely target genes near LINE-1s marked by repressive H3K9me3 only **(A)**, bivalent LINE-1s **(B)**, and LINE-1s with only the enhancer-associated marks **(C)**. The top 20 terms are shown, and their p values are plotted. Both enhancer-like and bivalent LINE-1s, but not repressed LINE-1s were enriched near genes involved in immune cell functions in these naïve B cells.

**
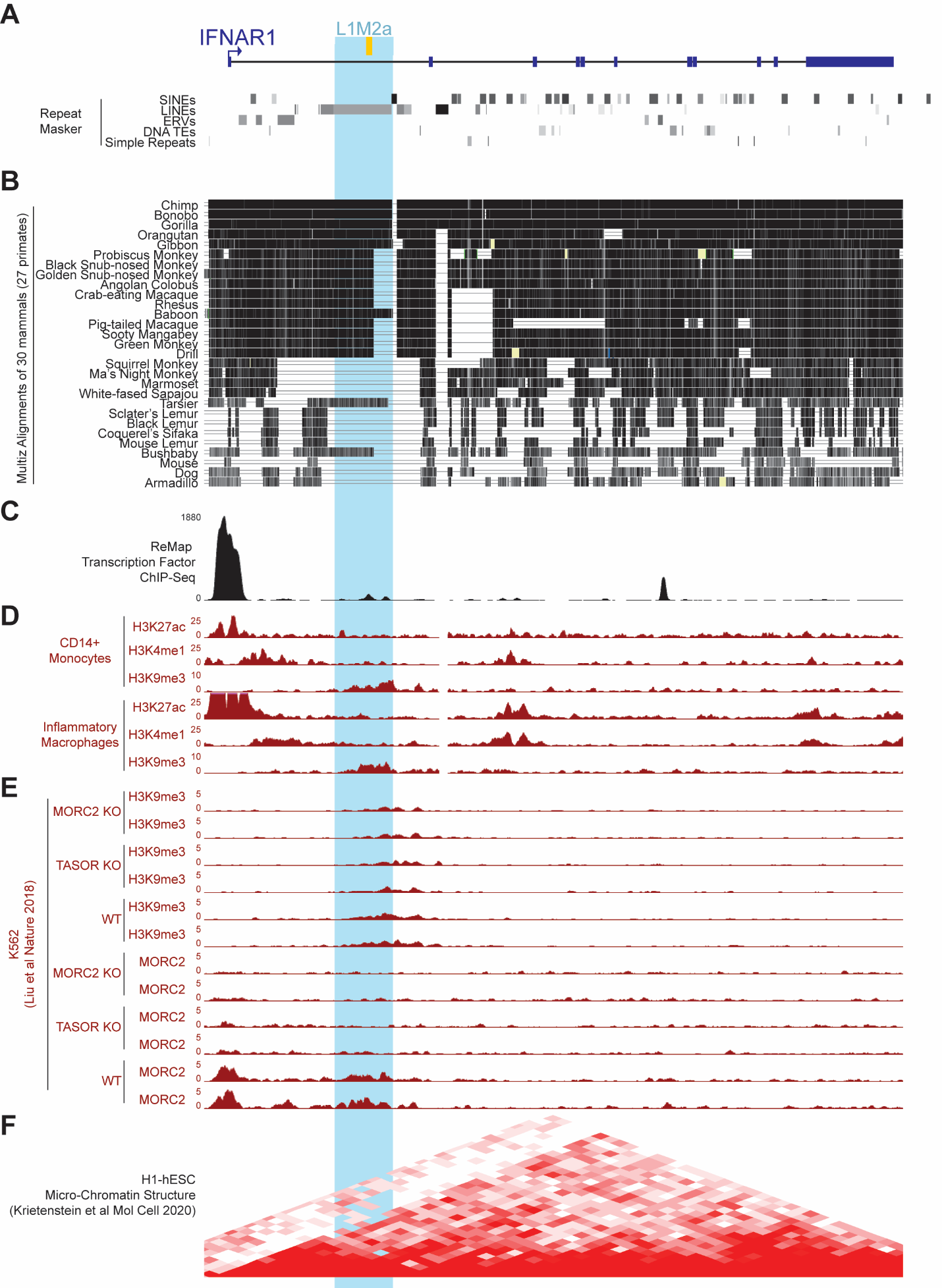
 Supplemental Figure 4. The IFNAR1 gene locus. (A)** The IFNAR1.L1M2a element (light blue) lies within the first intron of the IFNAR1 gene, and includes a predicted distal enhancer[42,65] **(B)** Evolutionary conservation of the IFNAR1.L1M2a element[41]. **(C)** Transcription factor binding peaks at the predicted enhancer[43]. **(D)** ChIP-seq data from primary human monocytes and macrophages[28] shows that not all immune cell types exhibit bivalent epigenetics at this locus. **(E)** Liu et al collected ChIP-seq data in their study[14] which shows that H3K9me3 overlap with the L1M2a element is dependent on the function of the HUSH complex, including the MORC2 and TASOR proteins.

**
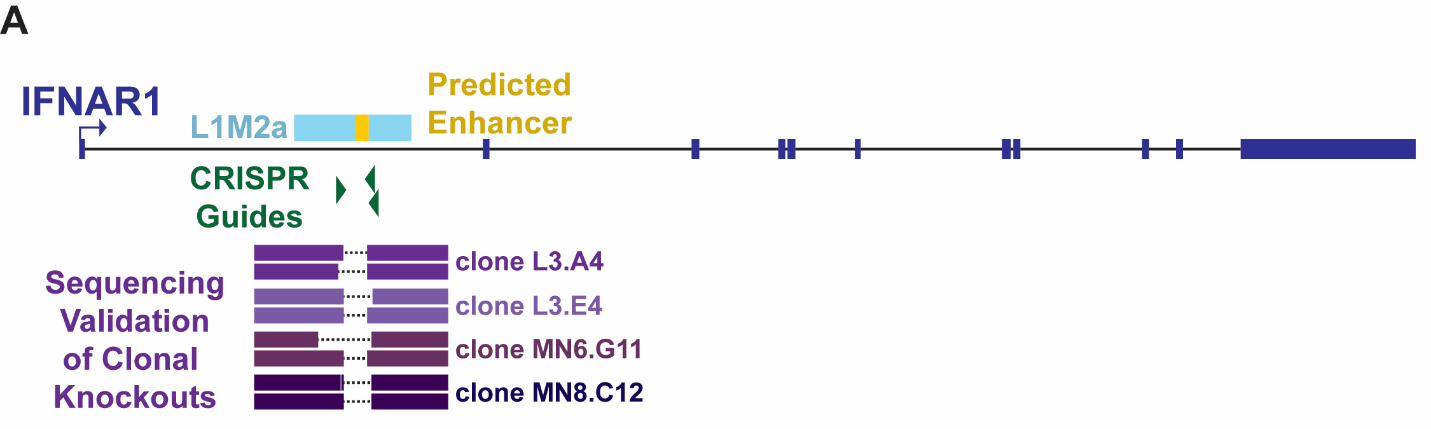
 Supplemental Figure 5. Sequencing validation of CRISPR knockout of IFNAR1.L1M2a.enh.** Four clonal lymphoblastoid cell lines (L3.A4, L3.E4, MN6.G11, and MN8.C12) were isolated with homozygous CRISPR knockout of IFNAR1.L1M2a.enh. The knockout regions were validated by sanger sequencing, and the alleles are displayed here, with the deleted regions marked with dotted lines.

**
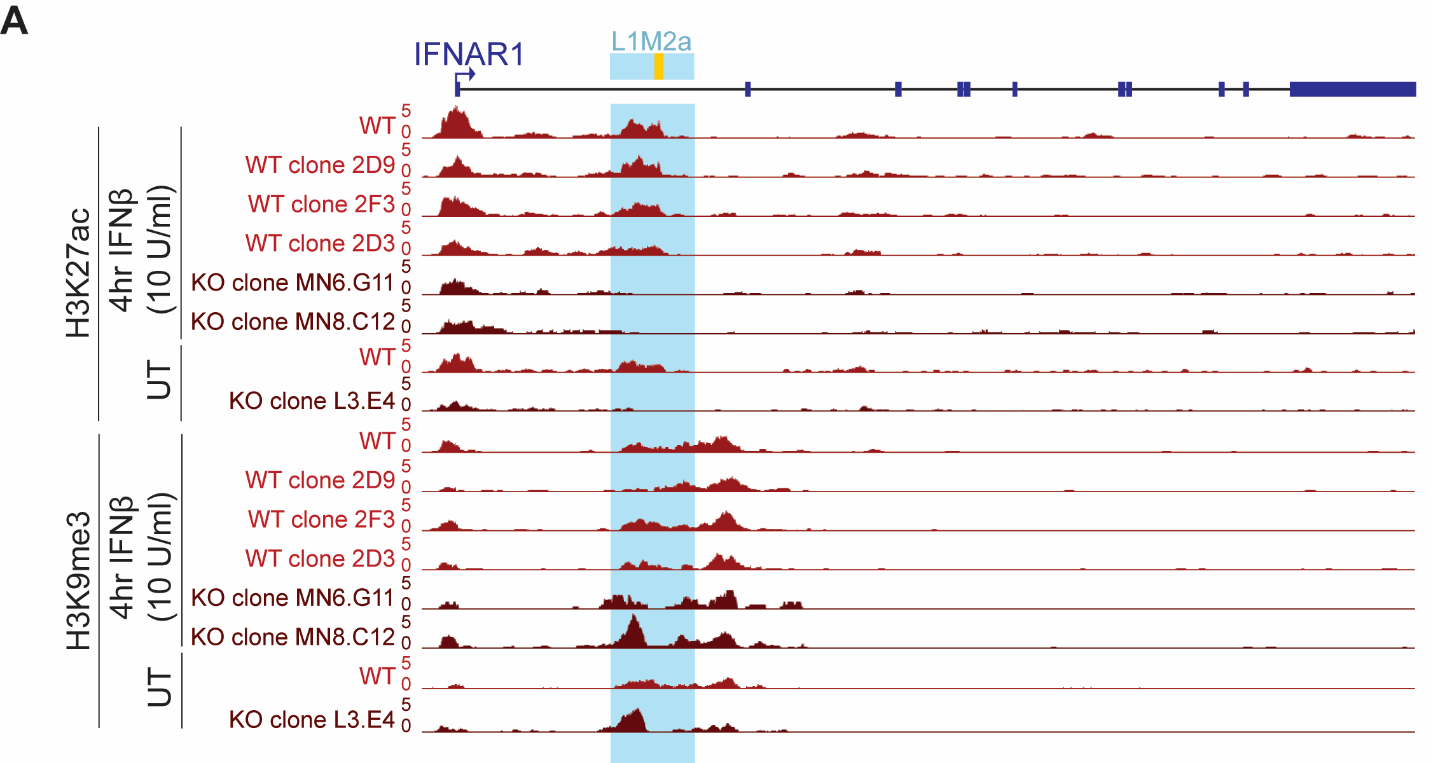
 Supplemental Figure 6. Chromatin profiling of the IFNAR1.L1M2a locus upon IFNβ signaling.** CUT&Tag[46] was used to collect ChIP-seq of active H3K27ac (top) and repressive H3K9me3 (bottom) in wild type (light red) and knockout (dark red) lymphoblastoid cells in untreated conditions and upon treatment with IFNβ for 4 hours.


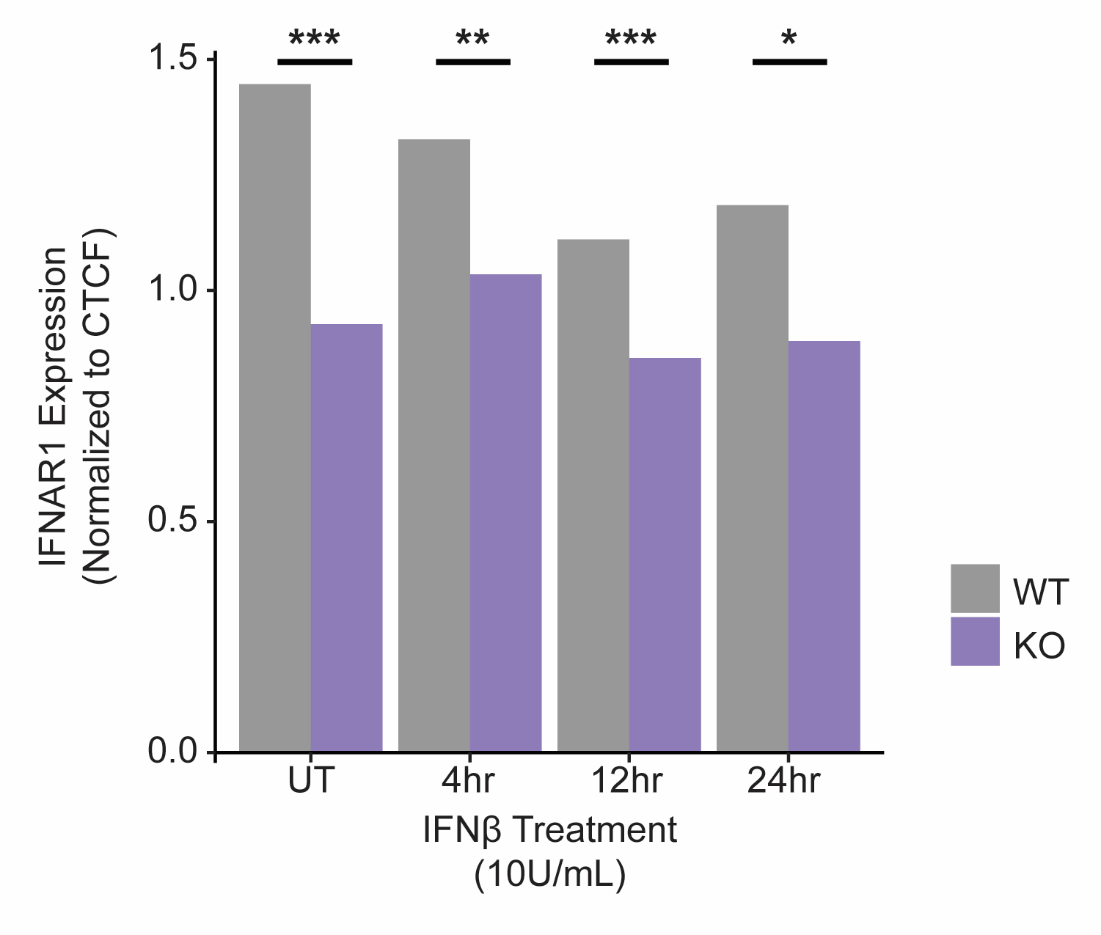


**Supplemental Figure 7. Quantification of IFNAR1 expression by RT-qPCR.** RT-qPCR was used in parallel with RNA-Seq to assess changes in the transcription of IFNAR1 upon IFNAR1.L1M2a.enh knockout. Significantly lower expression of IFNAR1 was observed in knockout cells (purple) compared with wildtype (gray) at all timepoints, using a student’s t-test. * indicates p<0.05. ** indicates p<0.01. *** indicates p<0.001.


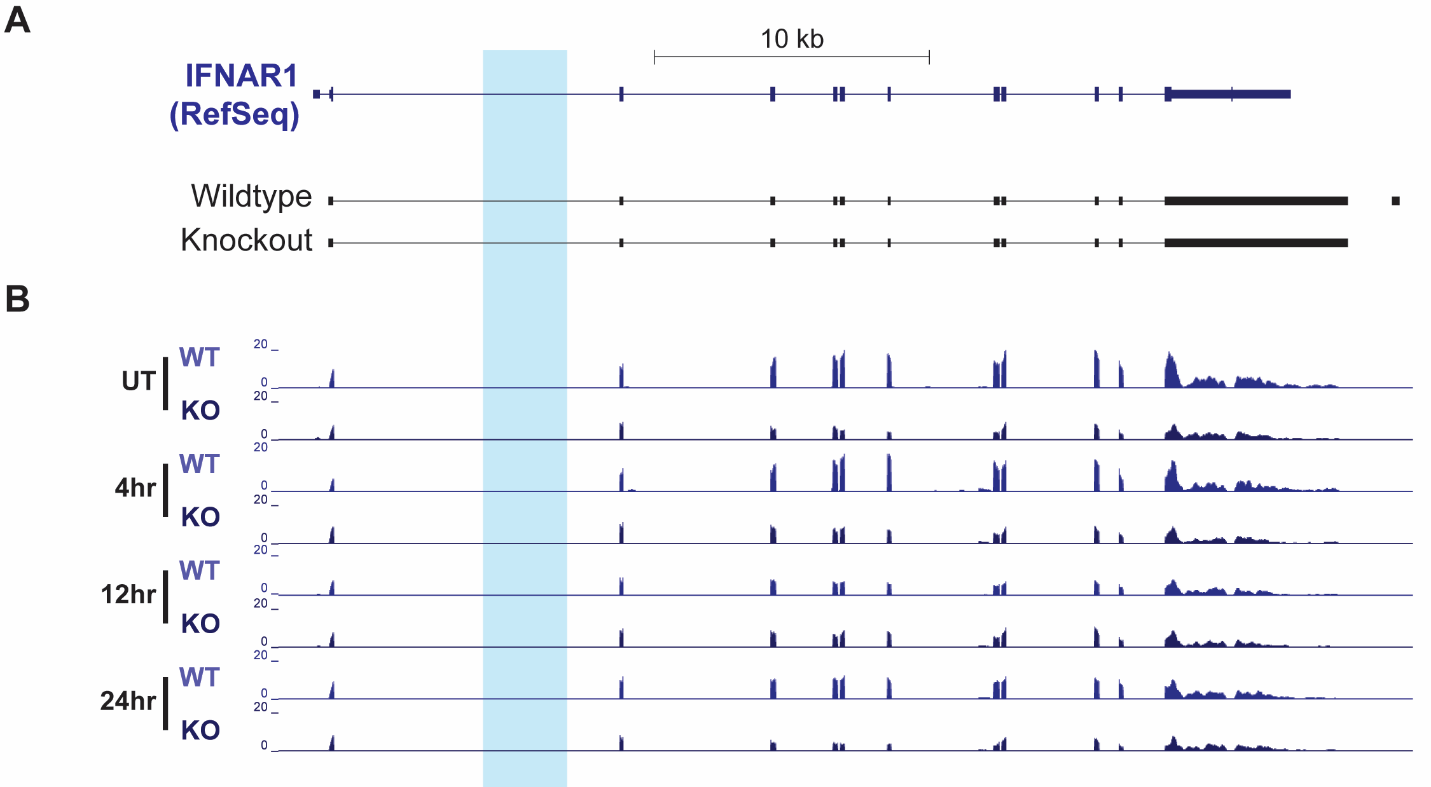


**Supplemental Figure 8. RNA-Seq and transcript assembly at the IFNAR1 locus. (A)** Transcript assembly using Stringtie[84] shows no differential splicing between wildtype and knockout cells at the IFNAR1 gene. **(B)** Buildup of RNA-Seq reads across the IFNAR1 gene in wildtype (light blue) and knockout (dark blue) cells from representative datasets (wildtype clone 2D3 and knockout clone L3.A4).

**
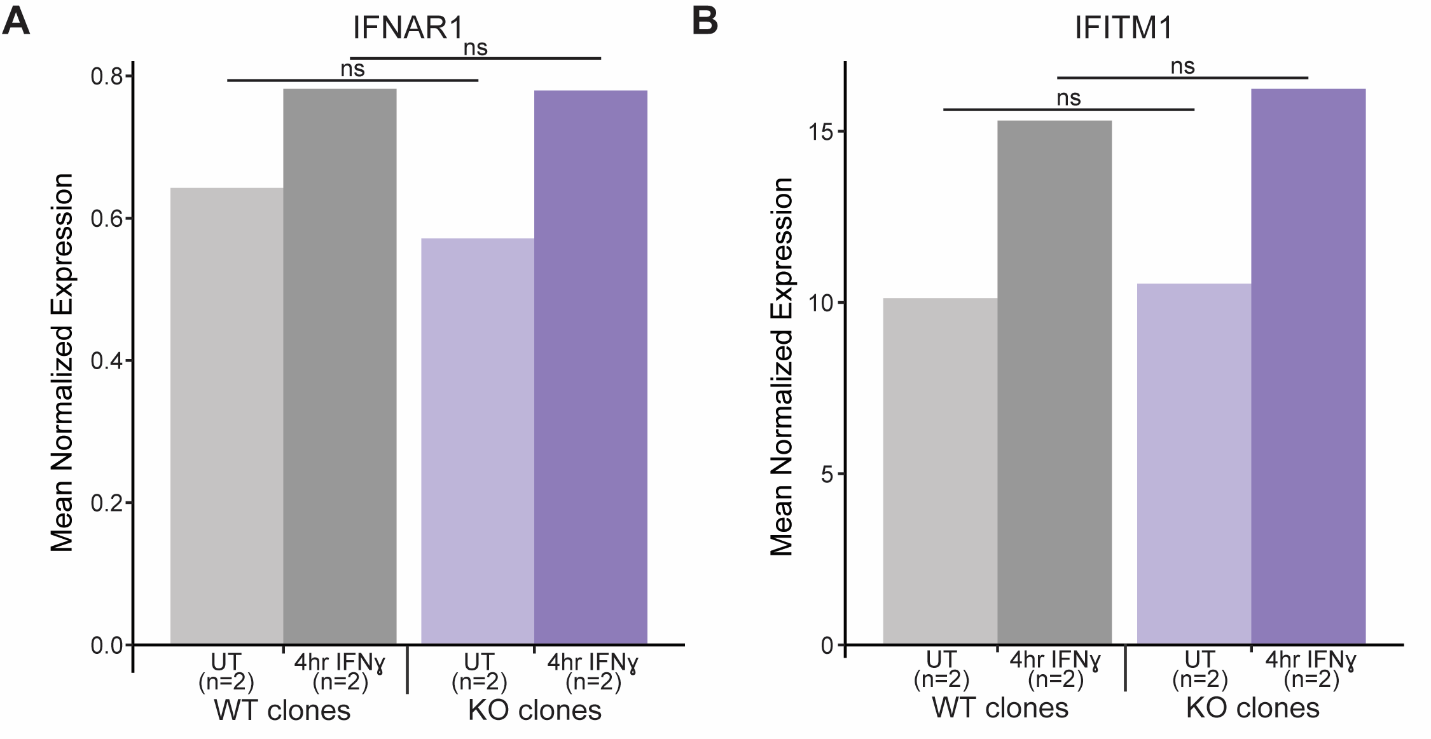
 Supplemental Figure 9. Transcriptional Response of wildtype and knockout cells to IFNɣ.** Quantitative PCR measuring expression, normalized to CTCF, of IFNAR1 **(A)** and representative IFN stimulated gene IFITM1 **(B)**, in wildtype compared with IFNAR1.L1M2a.enh knockout cells, under untreated and IFNɣ treated conditions. Two clonal cell lines of each genotype were used, each in duplicate. There was no significant difference between wildtype and knockout cells under the same treatment conditions.

**
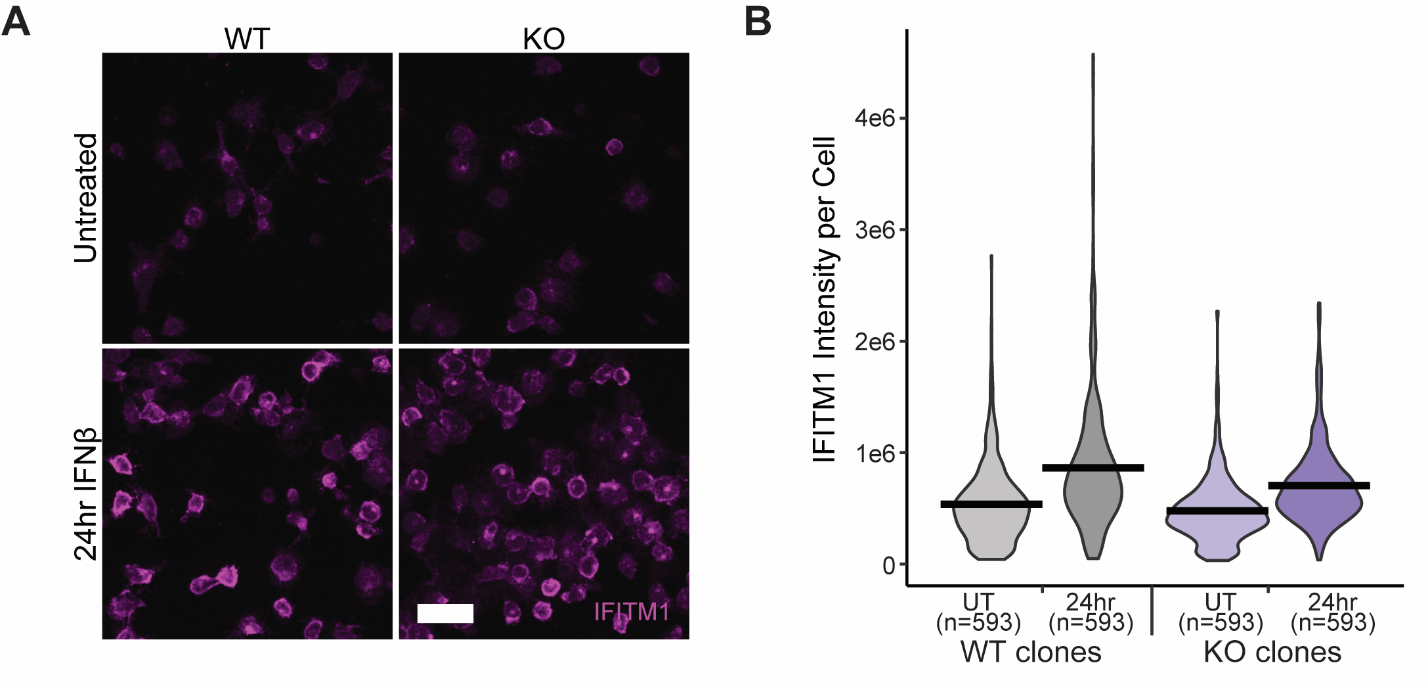
 Supplemental Figure 10. Immunofluorescence of IFITM1.** Immunofluorescence was used to approximate quantify IFITM1 protein abundance at the single cell level. **(A)** Representative images of IFITM1 labeling in wildtype and IFNAR1.L1M2a.enh knockout cells, under untreated and IFNβ-treated conditions. Scale bar is 25 microns. **(B)** Quantification of IFITM1 staining intensity per cell. Both wildtype and knockout cells show induction of IFITM1, though in knockout cells the induction appears less robust, in agreement with RNA-seq data (Fig. 5).
